# Mechanochemical bistability of intestinal organoids enables robust morphogenesis

**DOI:** 10.1038/s41567-025-02792-1

**Published:** 2025-02-28

**Authors:** Shi-Lei Xue, Qiutan Yang, Prisca Liberali, Edouard Hannezo

**Affiliations:** 1 Department of Materials Science and Engineering, School of Engineering, Westlake University, Hangzhou, China; 2 Institute of Science and Technology Austria, Klosterneuburg, Austria; 3 Friedrich Miescher Institute for Biomedical Research (FMI), Basel, Switzerland; 4 State Key Laboratory of Stem Cell and Reproductive Biology, Institute of Zoology, Chinese Academy of Sciences, Beijing, China; 5 Institute for Stem Cell and Regeneration, Chinese Academy of Sciences, Beijing, China; 6 Beijing Institute for Stem Cell and Regenerative Medicine, Beijing, China; 7 University of Chinese Academy of Sciences, Beijing, China; 8 Key Laboratory of Organ Regeneration and Reconstruction, Chinese Academy of Sciences, Beijing, China; 9 University of Basel, Basel, Switzerland

**Keywords:** Biological physics, Computational biophysics

## Abstract

Reproducible pattern and form generation during embryogenesis is poorly understood. Intestinal organoid morphogenesis involves a number of mechanochemical regulators such as cell-type-specific cytoskeletal forces and osmotically driven lumen volume changes. It is unclear how these forces are coordinated in time and space to ensure robust morphogenesis. Here we show how mechanosensitive feedback on cytoskeletal tension gives rise to morphological bistability in a minimal model of organoid morphogenesis. In the model, lumen volume changes can impact the epithelial shape via both direct mechanical and indirect mechanosensitive mechanisms. We find that both bulged and budded crypt states are possible and dependent on the history of volume changes. We test key modelling assumptions via biophysical and pharmacological experiments to demonstrate how bistability can explain experimental observations, such as the importance of the timing of lumen shrinkage and robustness of the final morphogenetic state to mechanical perturbations. This suggests that bistability arising from feedback between cellular tensions and fluid pressure could be a general mechanism that coordinates multicellular shape changes in developing systems.

## Abstract (web summary)

How forces are temporally coordinated during embryo development is unclear. Now two types of morphology are possible in a developing organoid and the final morphology depends on the system history.

## Main

Embryos are sculpted by a variety of physical forces^1–3^, such as active tensions from the cellular cytoskeleton^4^, and compressive stresses from external physical constraints or differential tissue growth^5–10^. Furthermore, hydrostatic pressure forces are emerging as important regulators for supracellular morphogenesis^11–22^. Although physical forces play key functional roles during morphogenesis, it is still unclear whether and how they are coordinated with each other and with concomitant biochemical signalling events, such as morphogen gradients and cell fate specification^23–26^. Indeed, cells and tissues typically experience large noise, both at the biochemical and mechanical levels, begging the question of how morphogenesis can occur in a reproducible manner^27–34^.

**Fig. 1:**
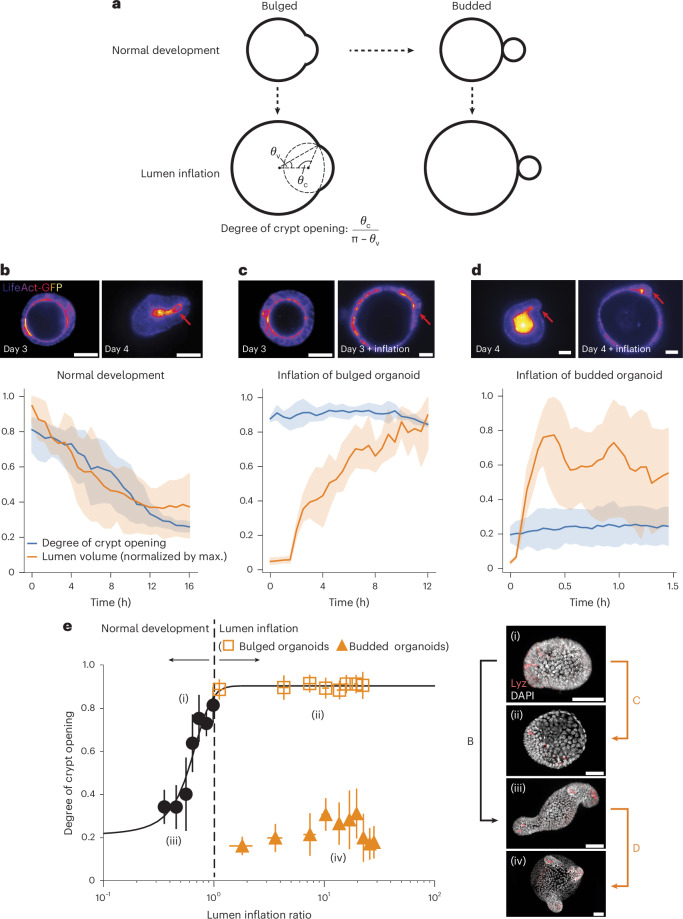
Crypt morphology depends on lumen volume changes in a history-dependent manner. **a**, Cartoon representation of normal and mechanically perturbed crypt morphogenesis. **b**–**d**, Top, representative time-lapse recordings of the normal development (**b**) of intestinal organoids, as well as the lumen inflation (performed by PGE treatment) of both bulged (**c**) and budded (**d**) organoids. Bottom, corresponding degree of crypt opening and lumen volume (normalized by the maximum value) as a function of time (number of samples *N* = 4 in each scenario). The solid lines represent the mean values and the shaded regions represent the 95% confidence intervals. **e**, Left, bistable relationship between the degree of crypt opening and lumen inflation ratio: early lumen inflation (**c**; (i)→(ii)) impairs long-term crypt budding morphogenesis, whereas lumen inflation after normal development (**d**; (iii)→(iv)) has negligible effect (number of samples *N* = 4 in each scenario). Data are presented as mean values ± s.d. Right, representative snapshots of four types of crypt state: (i) day 3.5 bulged organoid; (ii) day 4 organoid that remained bulged due to long-term lumen inflation; (iii) day 4 budded organoid (unperturbed); and (iv) day 4 budded organoid after lumen inflation. Images are the maximum *z* projections of the organoids with DAPI and Lyz staining. Scale bars, 50 µm (**b**–**e**). Source data

**Extended Data Fig. 1:**
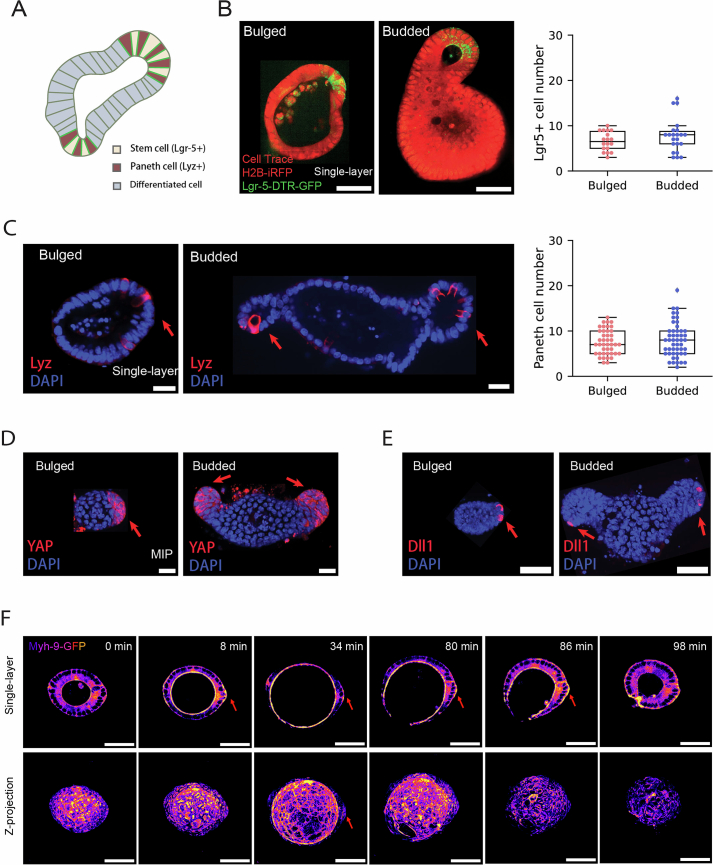
Cell-type composition in crypts and the relocation of actomyosin. **A**. Cartoon to show the cell types in intestinal organoid. **B**. Left, representative images for the straining of Lgr5 in both bulged and budded crypts. Cell trace indicates all cells, H2B-iRFP marks cell nuclei and Lgr5-DTR-GFP marks intestinal stem cells. Right, Lgr5^+^ cell numbers are similar in bulged and budded crypts (sample number *N* = 18 (bulged) and *N* = 22 (budded), p = 0.22, two-tailed t-test). **C-E**. Representative images for the staining of Lyz (C), YAP (D) and Delta 1(E) in crypts. Paneth cell numbers are similar in bulged and budded crypts (sample number *N* = 42(bulged) and *N* = 49 (budded), p = 0.48, two-tailed t-test). **F**. Time-lapse recordings of basal crypt actomyosin relocation during lumen inflation. Red arrows point to the crypt basal surface with increasing Myh-9-GFP intensity. Scale bars: 50 µm. The box-plot elements (in B and C) show the 25% (Q1, lower bound), 50% (median, line within the box) and 75% (Q3, upper bound) quartiles, and the whiskers denote 1.5× the interquartile range (minimum = Q1–1.5$\times$(Q3–Q1), maximum = Q3$+$1.5$\times$(Q3–Q1)) with outliers (rhombuses). Source data

The development of the vertebrate intestine represents a prototypical example of a complex morphogenetic sequence resulting in a highly stereotypical folded configuration, which is necessary to supply an adequate surface area for nutrient absorption^8,9^. The epithelium of the small intestine is organized in a folded monolayer with highly curved invaginations (crypts), where stem cells reside, and large finger-like protrusions (villi) into the intestinal lumen consisting of differentiated cells^9,35^. To overcome the limited accessibility of internal tissues, intestinal organoids have emerged as an ideal in vitro self-organized model system amenable to live imaging and mechanochemical perturbations^22,36–38^, particularly as organoid crypts display similar shapes and cell fates as their in vivo counterparts^37,39^ (Extended Data Fig. 1a–e). Organoid morphogenesis is organized by fate-dependent forces from the crypt and villi regions, actomyosin-driven apical constriction and lumen osmotic forces^22,36,40^ (Fig. 1a,b, Extended Data Fig. 1 and Supplementary Video 1).

**Extended Data Fig. 2:**
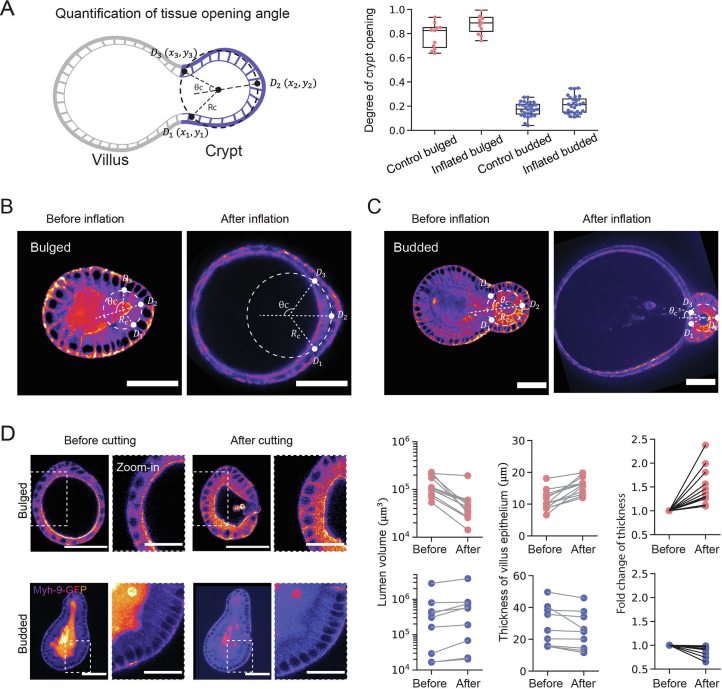
Quantification of tissue morphology and lumen volume. **A**. Quantification of crypt opening degree. Left, schematic to show data extraction for quantifying tissue opening angle (see Methods for details). Right, degree of crypt opening before and after lumen inflation, in both bulged (*N* = 11) and budded (*N* = 28) organoids. The box-plot elements show the 25% (Q1, lower bound), 50% (median, line within the box) and 75% (Q3, upper bound) quartiles, and the whiskers denote 1.5× the interquartile range (minimum = Q1–1.5$\times$(Q3–Q1), maximum = Q3$+$1.5$\times$(Q3–Q1)) with outliers (rhombuses). **B, C**. Quantification details of representative bulged and budded samples before and after lumen inflation. **D**. Quantification of lumen volumes before and after villus tissue breakage, in both bulged (top panels, *N* = 11) and budded (bottom panels, *N* = 9) organoids. Epithelial thickness in villus (absolute values and fold changes) can also infer the lumen volume changes: bulged samples show increased thickness of villus tissue after tissue breakage (top), indicating lumen volume decrease that is consistent with the volume measurement. Budded ones show the reverse trend (bottom). Scale bars: 50 µm (organoid) and 25 µm (magnification). Source data

**Extended Data Fig. 3:**
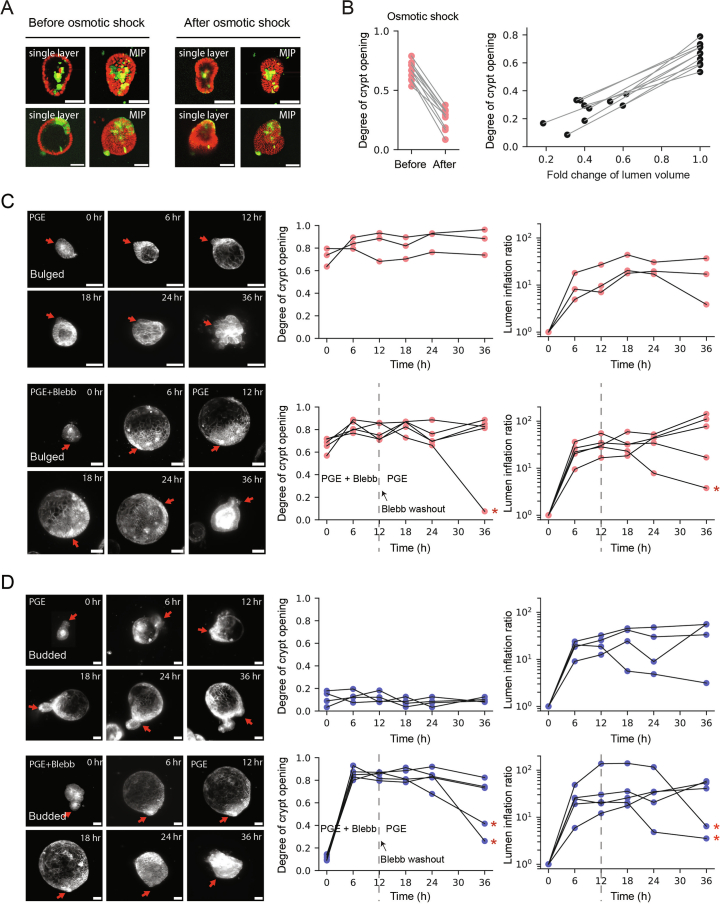
Crypt morphogenesis with various mechanical perturbations. **A**. Representative images of organoid before and after osmotic shock. **B**. Quantification of the degree of crypt opening and fold changes of lumen volume before and after osmotic shock (*N* = 10). **C, D**. Representative time-lapse images (left), evolution of the degree of crypt opening (middle) and the lumen volume (right) of bulged (C) or budded (D) organoids with two types of treatments: i) PGE treatment alone for 36 hours (see the upper panels in C and D, with *N* = 3 for bulged samples, and *N* = 4 for budded samples); ii) PGE and Blebbistatin treatments for 12 hours, followed by PGE treatment alone (Blebbistatin being washed out) for another 24 hours (see the lower panels in C and D, with *N* = 5 for both bulged and budded samples). Red arrows indicate the crypts. The red stars next to the last datapoints indicate the samples that show lumen deflate in the late stage, which correlate with late budding. Scale bar: 50 µm. Source data

However, whether these two key mechanical events are independently regulated or intrinsically coupled at the level of an entire organoid remains unclear. For instance, in our previous modelling, lumen deflation accelerates crypt budding, but is not strictly necessary for budding as long as apical actomyosin constriction is large enough. This is consistent with our previous data showing that budded crypts have morphologies robust to lumen volume increase^22^. Nevertheless, this does not explain our experimental observation that lumen volume reduction at the onset of morphogenesis is required for crypt budding (Fig. 1a–d, Extended Data Figs. 2 and 3 and Supplementary Videos 1–3). From a more conceptual level, this hints at the non-intuitive idea that the timing of a given mechanical event critically modulates its morphogenetic impact, with the same change in lumen volume giving rise to completely different outcomes if performed before or after crypt budding, despite similar cell fate compositions (Extended Data Fig. 1a–e). Such feature, not captured by current theoretical models, could act as a source of robustness for morphogenesis, as it would result in the crypt shape being both sensitive to mechanical forces during morphogenesis and insensitive to mechanical fluctuations once morphogenesis is completed.

Here we propose a biophysical theory for the coordination and robustness of mechano-osmotic forces driving intestinal organoid morphogenesis. Via an analytically tractable three-dimensional (3D) vertex model, we find that the phase diagram for organoid morphologies contains a mechanically bistable region: both open (bulged) and closed (budded) crypt configurations are possible for a specific organoid volume, and the ultimate morphological outcome depends on the history of the system. Biochemical bistability has been proposed as a key source of robustness for stem cell fate determination by allowing cells to both commit to a given fate on an inducing signal and retain this fate even after the signal has been removed^41,42^. We hypothesized that lumen pressure could play the role of an inducing signal in multicellular systems, with mechanical bistability resulting in organoid shape being both (1) tightly controlled by lumen pressure during morphogenesis and (2) highly insensitive to mechanical changes once morphogenesis is complete (Fig. 1a,e). We also find that the regime of parameters allowing for bistability is drastically enhanced by considering a mechanosensitive coupling between actomyosin tension and lumen pressure, which we verified experimentally and allowed us to quantitatively recapitulate organoid morphogenesis and various perturbations.

## Theory of bistable crypt morphology controlled by lumen volume

**Fig. 2:**
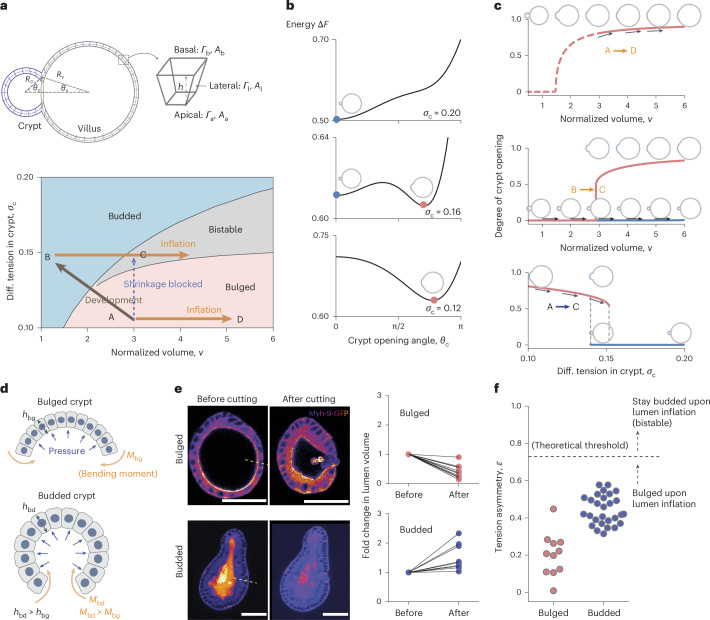
Theory of bistable crypt morphology switched by lumen volume. **a**, Schematic of a 3D vertex model (top) and the phase diagram of crypt morphology (bottom) as a function of crypt differential tension *σ*_c_ and normalized volume *v*. In the schematic, *R*_c_ and *R*_v_ are the radii of curvature in the crypt and villus, respectively; *h* is the cell height/tissue thickness (Supplementary Note 1.1). **b**, Three possible energy landscapes that control the crypt morphology (*v* = 4). Local energy minima are crypt equilibrium states, with *θ*_c_ = 0 indicating the budded (or closed) crypt shape and *θ*_c_ > 0 indicating the bulged (or open) shape. **c**, Evolution of crypt shape with varying lumen volumes at constant differential tension *σ*_c_ = 0.1 (top) and *σ*_c_ = 0.15 (middle), and evolution with varying crypt differential tension at a constant volume of *v* = 3 (bottom). The arrows are different paths shown in the phase diagram in **a**. **d**, Schematic of the mechanism of curvature–thickness feedback in the crypt epithelium: epithelial bending increases its thickness *h* and the corresponding active bending moment *M* ∼ *σ*_c_*h*, which, in turn, facilitates crypt budding. **e**, Fold changes of lumen volume before and after villus tissue breakage, in both bulged (*N* = 11) and budded (*N* = 9) organoids. Scale bars, 50 µm. **f**, Tension asymmetry of the crypt apical versus basal surfaces in bulged (*N* = 11) and budded (*N* = 28) organoids, and the theoretical threshold (dashed line) for organoids to remain in a budded/bistable state on lumen inflation. Source data

We theoretically describe an intestinal organoid as a closed epithelial monolayer with two different regions—crypt and villus—which encapsulates an incompressible fluid lumen. We have previously shown that such a two-region 3D vertex model, which captures the effects of cell-scale active forces on tissue-scale deformation, can accurately predict organoid morphologies^22^. In the model, we consider a single cell with three surface tensions *Γ*_a_, *Γ*_b_ and *Γ*_l_, arising from cell–cell adhesion and actomyosin-induced tension along the cell membrane^43–46^, and three surface areas *A*_a_, *A*_b_ and *A*_l_, with subscripts a, b and l representing the apical, basal and lateral surfaces/domains, respectively (Fig. 2a). Then, the free energy of a single cell reads

$$f={\varGamma }_{{\rm{a}}}{A}_{{\rm{a}}}+{\varGamma }_{{\rm{b}}}{A}_{{\rm{b}}}+\frac{1}{2}{\varGamma }_{{\rm{l}}}{A}_{{\rm{l}}}.$$

When considering that the crypt and villus regions can each have different mechanical properties, the possible morphologies of an organoid depend on four dimensionless parameters (Supplementary Note 1.1): (1) the relative size of the crypt *φ* (that is, the ratio of the number of crypt cells to the total number); (2) normalized organoid volume *v*; (3) in-plane tension ratio $\alpha =\frac{{\left({\varGamma }_{{\rm{a}}}+{\varGamma }_{{\rm{b}}}\right)}_{{\rm{c}}}}{{\left({\varGamma }_{{\rm{a}}}+{\varGamma }_{{\rm{b}}}\right)}_{{\rm{v}}}}$ (with the subscripts c and v denoting the crypt and villus tissues, respectively); and (4) differential tension between the apical and basal sides of the crypt cells ${\sigma }_{{\rm{c}}}=\frac{1}{2}{\left(\frac{{\varGamma }_{{\rm{a}}}-{\varGamma }_{{\rm{b}}}}{{\varGamma }_{{\rm{l}}}}\right)}_{{\rm{c}}}\sqrt{\frac{4\uppi }{{N}_{{\rm{t}}}}}$ (with *N*_t_ being the total number of cells in the organoid), which causes the crypt cells to have a preferred curvature.

**Extended Data Fig. 4:**
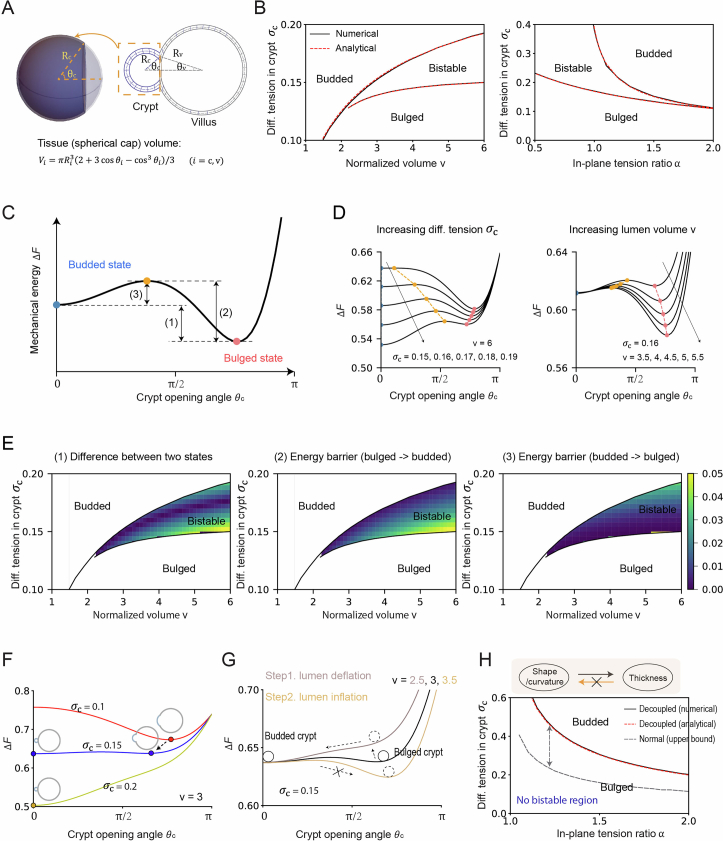
Detailed theoretical analysis of crypt morphological bistability without mechano-sensation. **A**. Three-dimensional geometry of intestinal organoid, and basic geometric relations used to establish the model (see SI Note section 1.1 for details). **B**. Comparison between numerical (from the full model) and analytical (from the simplified model) results of phase boundaries in ${\sigma }_{{\rm{c}}}-v$ diagram ($\alpha =1.2$, left panel) and ${\sigma }_{{\rm{c}}}-\alpha$ diagram ($v=10$, right panel), showing high consistency. **C**. Schematics of a bistable energy landscape with mechanical energy $\Delta F$ as a function of crypt opening degree ${\theta }_{{\rm{c}}}$. Two local minima respectively correspond to budded (${\theta }_{{\rm{c}}}=0$) and bulged (${\theta }_{{\rm{c}}} > 0$) crypt shapes, and we label (1) the energy difference between bulged and budded states, (2) the energy barrier for bulged to budded transition, and (3) the energy barrier for budded to bulged transition. **D**. Evolution of energy landscapes for varying differential tension ${\sigma }_{\tt{c}}$ (left) and lumen volume $v$ (right). **E**. Distributions of energy barriers in the bistable region of ${\sigma }_{{\rm{c}}}-v$ phase diagram. **F, G**. Variation of the energy landscape indicates the evolution of crypt shape with differential tension ${\sigma }_{{\rm{c}}}$ (F) and lumen volume $v$ (G). **H**. Crypt morphology in ${\sigma }_{{\rm{c}}}-\alpha$ diagram after decoupling crypt curvature and thickness, and comparison with the original model. Compared to panel B, this shows an absence of bistable region, as well as higher threshold for budding.

Previously, we considered apical myosin in crypts as the key parameter^22^, which changes both in-plane tension *α* and differential tension *σ*_c_. However, more complex regulatory patterns of myosin can be observed experimentally, including basal crypt actomyosin relocation during lumen inflation (Extended Data Fig. 1f and Supplementary Video 4). When theoretically investigating the separate consequences of differential tension *σ*_c_ and in-plane tension *α*, we found that in specific regions of the phase diagram (Fig. 2a), crypt morphology can show two stable configurations (either open or closed shape) for the same lumen volume and, thus, display morphological bistability. To gain insights into this theoretical phenomenon, we consider the limit of large lumen volume, for which the dependency of the total mechanical energy^47^ of the organoid on the crypt shape (represented by crypt opening angle *θ*_c_) can be derived analytically:

$$\Delta F(x)={\left[1-{\sigma }_{{\rm{c}}}{\left(\frac{1+x}{2\varphi }\right)}^{\frac{1}{2}}\right]}^{\frac{2}{3}}{\left(1-\frac{1-x}{2\alpha }\right)}^{\frac{1}{3}}\,,$$

where *x* = cos(*θ*_c_). Depending on differential tension *σ*_c_, three scenarios exist (Fig. 2b and Extended Data Fig. 4): (1) for small *σ*_c_, the crypt epithelium will stay open/bulged; (2) for intermediate differential tension *σ*_c_, two local energy minima exist, indicating that both open and closed configurations are possible; (3) for large enough *σ*_c_, the crypt will always be budded. Next, we look at the morphological evolution of the organoid shape on lumen volume change. The ‘degree of crypt opening’, described as *θ*_c_/(π – *θ*_v_), is used to quantify the crypt morphology (Figs. 1 and 2c and Extended Data Fig. 2), ranging from 0 to 1, with 0 standing for the budded shape (with the crypt and villus fully closed) and 1 for a spherical organoid. For small differential tension (for example, *σ*_c_ = 0.1), crypt morphology evolves continuously and monotonously with changes in the lumen volume: the crypt opens (or closes) on lumen inflation (or shrinkage) (characterized by path A→D). With intermediate differential tension (for example, *σ*_c_ = 0.15), the crypt gradually closes up with lumen shrinkage until fully closed; however, the morphological evolution is no longer reversible as this closed crypt cannot be opened by further volume inflation (path B→C).

Such morphological hysteresis—or path-dependent evolution—is a classical feature of bistable systems^41^, and is qualitatively consistent with our data (Fig. 1). Starting from a bulged/open crypt, increasing the differential tension at a constant volume drives an entry into the bistable region from the ‘bulged’ configuration. Therefore, crypts are prone to stay bulged (path A→C)—mirroring our data that impairing volume decrease during morphogenesis (day 3 to day 4) impairs crypt budding (Fig. 1c and Supplementary Video 2). By contrast, if the differential tension increases at the same time as the decrease in lumen volume (path A→B), organoids can reach the monostable budded state, consistent with our data on normal organoid morphogenesis (Fig. 1b and Supplementary Video 1). Crucially, the subsequently increasing lumen volume back to its initial value once crypts are already budded (path B→C) results in organoids entering the bistable region from the ‘budded’ configuration and, thus, remain in this state, as in our inflation data (Fig. 1d and Supplementary Video 3), despite reaching the same final volume as in the first path (A→C).

This suggests that the experimental system could exhibit morphological hysteresis and bistability as in the model, although this needed to be systematically tested. Before turning to a more quantitative comparison between data and theory, we proceeded to better understand the mechanical origin of bistability in the model.

## Mechanical origin of morphological bistability

Our 3D vertex model of intestinal organoids is conceptually related to the classical Helfrich theory for lipid vesicles with prepatterned curvatures ^48,49,50^, except for the key difference in morphological bistability observed above. However, we reasoned that unlike lipid membranes, epithelia have a comparatively large thickness, which changes with active tensions and deformations^22,36,51–54^.

During crypt morphogenesis, the out-of-plane deformation driven by differences in apicobasal tension tends to increase the epithelial thickness as the crypt curvature increases,^22,46,55,56^, and the driving force (‘active bending moment’) *M* arising from differential tension is also proportional to the epithelial thickness (that is, *M* ∼ (*Γ*_a_ – *Γ*_b_)*h*; Fig. 2d). Therefore, the epithelial thickness and out-of-plane deformation can have positive feedback: under this geometry, the bending tends to thicken the epithelium, which, in turn, enhances *M* and, thus, facilitates out-of-plane deformation. This effect saturates, as thicker tissues also have higher bending stiffness. To computationally test this hypothesis, we examined an alternative model in which the crypt thickness is kept constant, and confirmed that bistability was absent (Extended Data Fig. 4h and Supplementary Note 1.4). Overall, purely mechanical feedback can, thus, provide a potential explanation for crypt bistability and, thus, the history-dependent effects of lumen volume changes that we observed experimentally.

Next, we aimed to quantitatively test this hypothesis. We noticed that the proposed theoretical mechanism only works when the lumen of the organoid is initially swollen, that is, when the fluid pressure exerts tension on the epithelium (*v* > 1 in the phase diagram; Fig. 2a). We, thus, inferred the mechanical state of the lumen by experimentally measuring its volume before and after inducing epithelial breakage with 3D localized laser ablation of the tissue: if the lumen is initially swollen, tissue breakage will then let the luminal fluid flow out, and vice versa. Importantly, these measurements (Fig. 2e and Extended Data Fig. 2d) confirmed that bulged organoids are in the swollen state (*v* ≈ 2.77 ± 1.27, mean ± s.d.), whereas budded samples are slightly shrunk (*v* ≈ 0.71 ± 0.18)—consistent with the 20–80% decrease in lumen volume observed during normal organoid morphogenesis from the bulged to budded shapes^22^. Furthermore, we quantitatively reanalysed osmotic deflation experiments^22^ in terms of the crypt opening angle, and found that a 60% lumen volume decrease is sufficient to drive fast crypt budding, which is consistent with our inferred value for *v* (Extended Data Fig. 3a,b and Supplementary Note 1.3).

Importantly, however, with this experimentally inferred value of lumen volume, bistability is only predicted to occur in a very limited parameter range of tensions *σ*_c_ (0.14–0.15; Fig. 2a), which would require extreme fine tuning of cellular tensions in organoids. Given the natural variabilities in myosin and tension levels^22^, this scenario would fail to ensure the robustness of crypt morphogenesis. Furthermore, given previous findings that myosin intensity ratios can serve as a good proxy for tension ratios^57,58,59^, we quantified the intensities of the fluorescent reporter for the force-generating non-muscle myosin II isoform (Myh9-GFP) in the crypt apical and basal surfaces to calculate the in-plane-to-lateral tension ratio $\frac{{{\varGamma }_{{\rm{a}}}+\varGamma }_{{\rm{b}}}}{{\varGamma }_{{\rm{l}}}}$ and in-plane tension ratio *α* (Extended Data Figs. 7–9), as well as estimate the ‘tension asymmetry’ in the crypt apical and basal tensions, defined as $\epsilon =\frac{{{\varGamma }_{{\rm{a}}}-\varGamma }_{{\rm{b}}}}{{\varGamma }_{{\rm{a}}}+{\varGamma }_{{\rm{b}}}}$ (Supplementary Note 3 provides details on the quantifications and data-model comparisons). Importantly, we found that tension asymmetries $\epsilon$ in the budded crypts are below the theoretical threshold required for organoids to stay within the budded or bistable region of the phase diagram on lumen inflation (Fig. 2f and Supplementary Note 4.2). This discrepancy with our experimental data (Fig. 1a,e) indicates that although the model can qualitatively resolve the paradox of distinct lumen inflation effects at different time points, other mechanisms must be at play to ensure that organoids robustly remain in the bistable region of the phase diagram (rather than re-entering the ‘bulged crypt’ region) on mechanical perturbations.

## Morphological bistability with mechanosensitivity of crypts

**Fig. 3:**
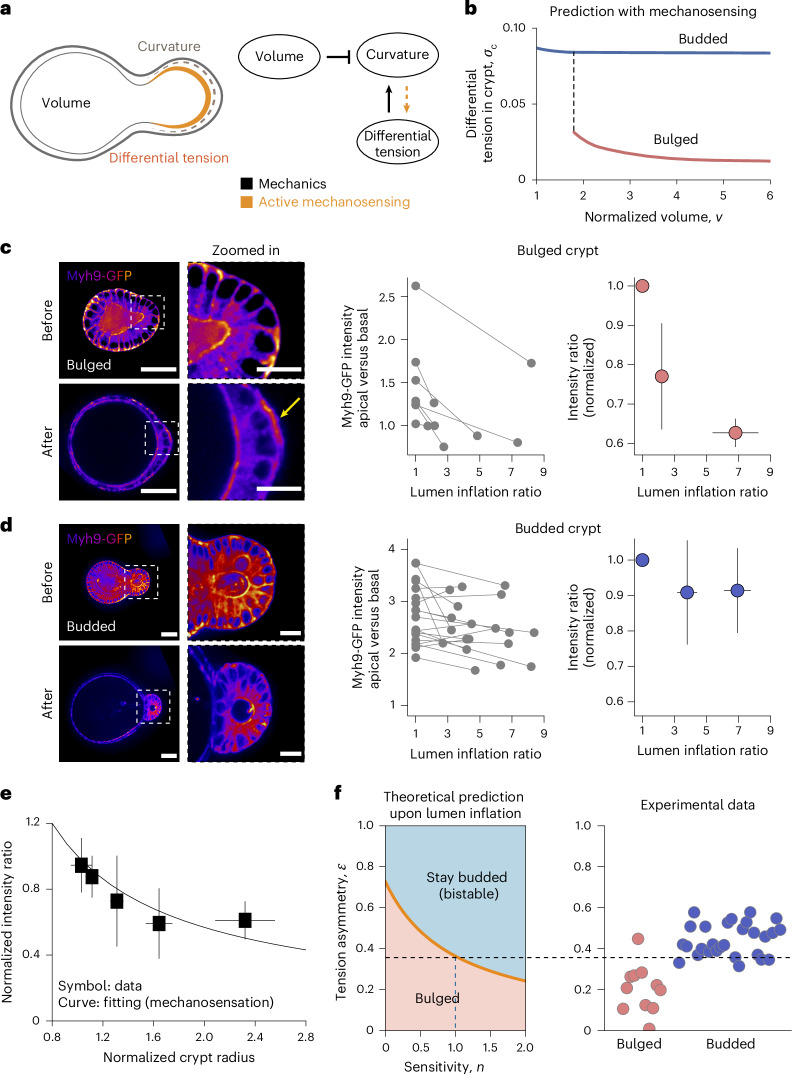
Morphological bistability with mechanosensitivity of crypts. **a**, Schematic of the feedback mechanism that involves both mechanical forces and mechanosensitivity of crypt cells: lumen volume and apical-to-basal tension difference affect the curvature of the crypt due to passive force balance, whereas active mechanosensing can result in the geometry/mechanics of the crypt feedback on apical/basal tensions. **b**, Theoretical prediction of the dependence of crypt differential tension on lumen volume, in both bulged and budded organoids, when assuming mechanosensation: in bulged organoids, inflation results in crypt opening that negatively regulates tensions by mechanosensing, whereas budded organoids have structurally stable crypts that do not open on inflation that, thus, do not trigger an active response. **c**,**d**, Left, Myh9-GFP distribution on crypt surfaces, before and after lumen inflation (with time interval between these two states being 15–30 min). After lumen inflation, bulged crypts show basal actomyosin relocation (yellow arrow). Right, apical-to-basal Myh9-GFP intensity ratio in bulged (**c**; *N* = 7) and budded (**d**; *N* = 19) crypt cells. Both raw data and mean values ± s.d. of data are shown. **e**, Experimental data (symbols, presented as mean values ± s.d.) and fitting (line) of mechanosensitivity, both apical-to-basal Myh9-GFP intensity ratio and crypt radius after lumen inflation are normalized by their values before inflation. Both bulged (*N* = 24) and budded (*N* = 28) samples are included. The fitting curve is *y* = *x*^–*b*^. **f**, Influence of sensitivity factor *n* on the predicted threshold for organoids to remain in the budded/bistable state on lumen inflation, and comparison with experimental data of bulged (*N* = 11) and budded (*N* = 28) samples for the estimated value of *n* = 1.0 (inferred from the best-fit value in **e**; see the main text and Supplementary Note 4 for details). Scale bars, 50 µm (organoid); 20 µm (zoomed-in image). Source data

**Extended Data Fig. 5:**
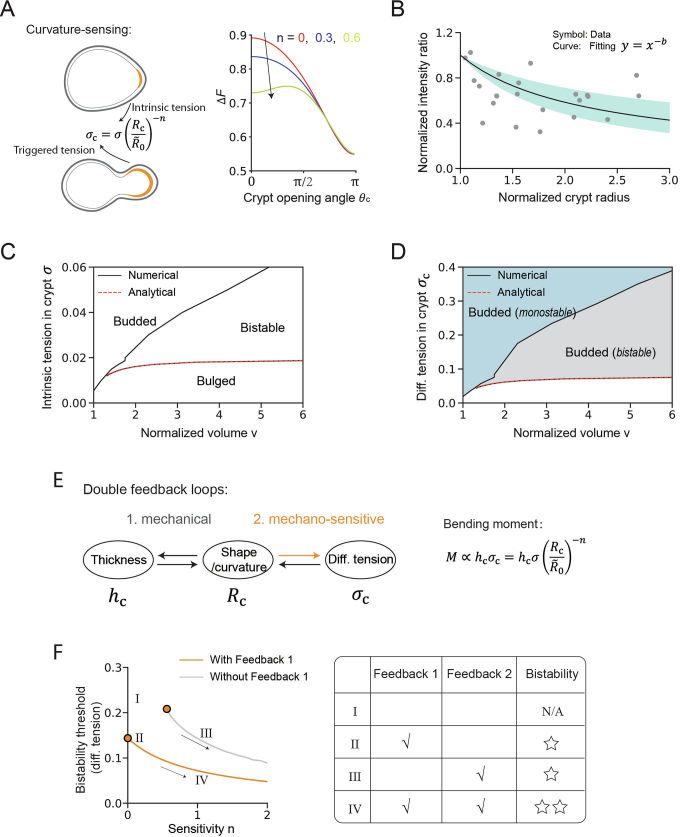
Detailed analysis of crypt morphological bistability with curvature-sensitivity. We set crypt size $\varphi =0.1$ and in-plane contraction ratio $\alpha =1.2$ for all theoretical predictions (based on quantifications in Extended Data Fig. 9 for $\alpha$ and $\varphi$, and see SI Note section 3.2 and 4.3 for details). **A**. Left, schematic of the curvature-sensing mechanism, where the total differential tension ${\sigma }_{{\rm{c}}}\,$ is the product of an intrinsic value $\sigma$ (arising from stem cell fate,^22^) as well as a contribution inversely proportional to the crypt radius of curvature ${R}_{{\rm{c}}}$ with sensitivity $n$. Right, influence of curvature-sensitivity ($n$) on the energy landscape ($\sigma =0.05$, infinite volume), showing that it gives rise to a second minimum. **B**. Fitting of curvature-sensitivity. Apical to basal Myh9–GFP intensity ratio and crypt radius after lumen inflation are normalized by their values before inflation (bulged samples, *N* = 24). Best fit of data and 95% confidence interval are respectively shown as solid line and shaded region. **C, D**. Crypt morphology with curvature-sensation ($n=1$) in $\sigma -v$ diagram (C) and corresponding ${\sigma }_{{\rm{c}}}-v$ diagram showing an enhanced region of bistability (D, compare to Fig. 2a and Extended Data Fig. 4B). **E**. Double feedback loops in crypt morphogenesis. Feedback 1 is the mechanical coupling between epithelial thickness and curvature (see Fig. 2d for schematic), and Feedback 2 is the coupling between crypt tension and deformation (with mechano-sensation, see panel A for schematic). **F**. Evaluation of specific feedback loops that enable crypt bistability (see SI Note section 2.3 for details). Left, bistability threshold of differential tension ${\sigma }_{{\rm{c}}}$ without/with the purely mechanical Feedback 1 as well as its dependence on Feedback 2 (mechano-sensation with sensitivity $n$). Right, summary of the effects of two feedback loops on the size of the bistable region in phase diagram. Source data

**Extended Data Fig. 6:**
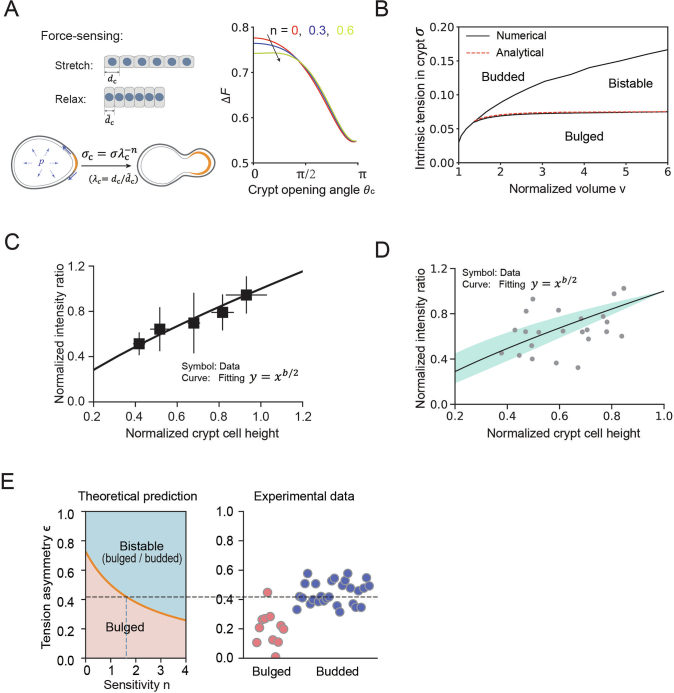
Crypt morphological bistability with force-sensitivity. **A**. Left, schematic of force-sensing mechanism, with crypt cell in-plane stretch ratio ${\lambda }_{{\rm{c}}}$ defined. Right, influence of force-sensitivity ($n$) on the energy landscape ($\sigma =0.1$, infinite volume), showing qualitatively similar features as curvature-sensing. **B**. Phase diagram of crypt morphology with force-sensation. **C, D**. Fitting of force-sensitivity. Apical to basal Myh9–GFP intensity ratio and crypt cell height (or thickness) after lumen inflation are normalized by their values before inflation (bulged samples, *N* = 24). The fitting curve is $y={x}^{b/2}\,$, with the fitting parameter $b$ used to infer force-sensitivity $n$. Fittings the mean values (squares, panel C) and raw data (dots, panel D) give similar best-fit values (see SI Note section 4.1 for details). Error bars (panel C) represent SD, the shaded region (panel D) represents 95% confidence interval. **E**. Predicted influence of sensitivity factor $n$ on the bistability threshold of tension asymmetry ${\epsilon }$, and comparison with experimental data of bulged (*N* = 11) and budded (*N* = 28) samples for the best-fit value $n=1.7$ from panel C. Source data

**Extended Data Fig. 7:**
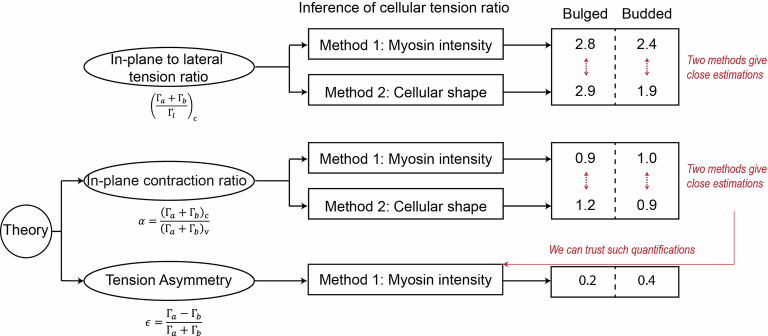
Quantification strategy of mechanical parameters related to cellular tension. Table showing the comparison of different quantification procedures.

So far, we have assumed that lumen volume changes and actomyosin tensions are fully independent parameters. However, the volume and pressure of the luminal fluid have a direct impact on the geometry and stresses of the crypt tissue, which could feed back on cellular surface tension via a variety of mechanosensitive mechanisms, as described in other model systems^60–63^. Furthermore, stem cells in crypts were found to be mechanosensitive as evidenced by the upregulation of Piezo1 and loss of stemness, on organoid inflation^38^. Recent in vitro experiments on substrates of well-defined geometries have also revealed that intestinal crypt formation can be biased by substrate curvature^64–66^, in line with a growing body of evidence of mechanosensation from tissue curvature^56,67,68^. On the basis of these findings, we considered two generic mechanisms of mechanotransduction: (1) stress-dependent feedback in which the mechanical stresses in the crypt modulates actomyosin tensions; or (2) curvature-/geometry-dependent feedback in which the curvature of the crypt cells influences actomyosin tensions. Given that both models give rise to qualitatively similar theoretical predictions (Extended Data Figs. 5 and 6 and Supplementary Note 2), we concentrate here on curvature-dependent feedback, as the curvature is easier to measure experimentally than stress (Fig. 3a; Supplementary Notes 2.2, 4.1 and 4.2 provide an alternative case). More specifically, we consider the equation ${\sigma }_{{\rm{c}}}=\sigma {\left(\frac{{R}_{{\rm{c}}}}{{\widetilde{R}}_{0}}\right)}^{-n}$, where the crypt differential tension *σ*_c_ depends on an intrinsic value *σ* (set by the stem cell fate^22^) and on the crypt radius of curvature *R*_c_ (normalized by a reference value before morphogenesis ${\widetilde{R}}_{0}$), with *n* being the coefficient quantifying the coupling strength.

Interestingly, such feedback functions distinctly in bulged versus budded crypts (Fig. 3b and Extended Data Fig. 5): decreasing the lumen volume in bulged organoids (as occurs in normal organoid development) causes an increase in the crypt differential tension, because it affects the crypt geometry. Thus, lumen deflation critically contributes to crypt morphogenesis, both by its direct mechanical impact (decreasing the monolayer tension) and indirect mechanosensitive consequence (increasing the actomyosin differential tension *σ*_c_). However, once the differential tension is sufficiently high such that the crypt becomes budded, as previously discussed (Fig. 2a–c), the system is mechanically ‘trapped’ in the budded shape as it becomes energetically more favourable to deform the villus cells, rather than the crypt cells, on inflation.

## Lumen volume changes affect actomyosin localization

To test these predictions, we first experimentally validated the key modelling assumption that differential tension is dependent on crypt geometry and lumen volume. Thus, we increased the lumen volume in early bulged or late budded organoids by treatment with prostaglandin E2 (PGE). We had previously shown that this results in crypt opening in bulged organoids, but no change in the budded crypt geometry^22^. Importantly, we found that these volume perturbations resulted in very different consequences on the levels and localization of myosin in bulged versus budded crypts. Although bulged crypts displayed lowered apical-to-basal myosin ratio on volume inflation in a dose-dependent manner (Fig. 3c), budded crypts did not show a consistent change (Fig. 3d). This argues that the changes in actomyosin localization in bulged crypts are not a secondary effect of osmotic changes on inflation, but are linked to changes in crypt geometry. Furthermore, inflation and myosin response occurred much faster (15–30 min; Fig. 3c,d) than the timescales of cell fate or lumen volume change during morphogenesis, justifying our quasistatic theoretical setting (Fig. 3b; Extended Data Fig. 10a–c and Supplementary Note 5 provide a full consideration of different timescales).

**Fig. 4:**
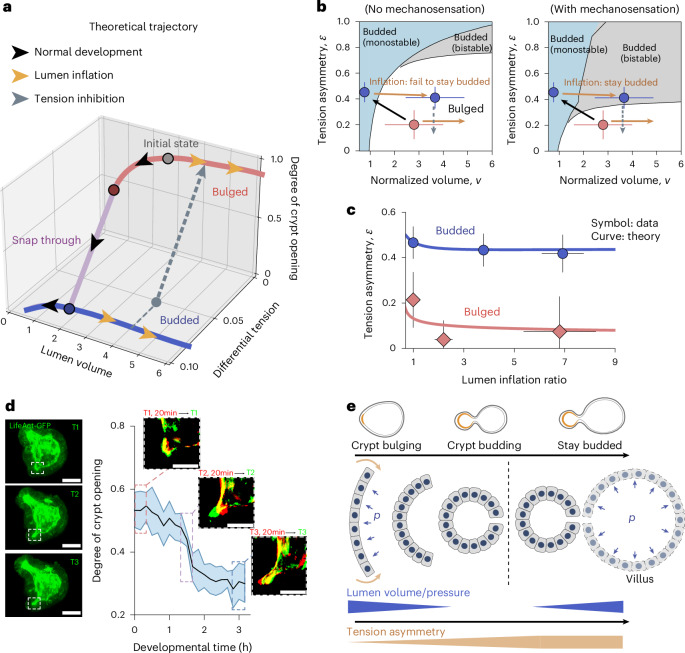
Theoretical predictions of mechanochemical bistability for crypt morphogenesis. **a**, Theoretical bifurcation trajectories showing the evolution of crypt morphology and differential tension with lumen volume, specified as normal morphogenesis (black arrows), lumen inflation of both bulged and budded organoids (orange arrows), and tension inhibition (grey arrows). **b**, Comparison between experimental data (red dot, bulged (*N* = 11) samples; blue dots, budded (*N* = 28) samples; all data presented as mean ± s.d.) and predicted phase diagrams of crypt morphology as a function of tension asymmetry $\epsilon$ and normalized volume *v*. Tension asymmetry of budded organoids agrees with the prediction considering crypt mechanosensation (right) rather than that without mechanosensation (left; see also Fig. 2f). **c**, Predicted evolution for tension asymmetry data (mean ± s.d.) of both bulged (*N* = 7) and budded (*N* = 19) samples with the theoretical model, where the intrinsic differential tension *σ* is the only free parameter fitted as 0.02 (Supplementary Note 4.3). **d**, Evolution of crypt shape with developmental time (*N* = 5), showing rapid changes in shape at a critical morphological point, as expected from bistability. The solid line represents mean values and the shaded region represents 95% confidence intervals. For different developmental stages, changes in the crypt profiles in a time interval of 20 min (red, start of interval; green, end of interval) are shown. Scale bars, 50 µm. **e**, Schematic of crypt morphogenesis driven by luminal pressure, and involved morphological bistability feature arising from mechanosensation. Source data

Importantly, across all the bulged and budded inflation data, we found a consistent trend linking the relative actomyosin intensity to crypt curvature (Fig. 3e). We then used this general relationship to quantitatively parameterize the mechanosensitive feedback between apicobasal tensions and geometry in the model (that is, $\bar{\epsilon }={\bar{R}}_{{\rm{c}}}^{-n}$), leading to a fit for the mechanosensitivity factor of *n* = 1.0 ± 0.5 (Supplementary Note 4.1). On incorporation of this mechanosensitive coupling parameter, the theory predicted much broader regions of shape bistability (Extended Data Fig. 5c,d; for instance, at *v* = 3, bistability occurs when *σ* is between 0.02 and 0.04—a range that is an order of magnitude broader than without mechanosensing), which allows for a robust and irreversible budding of crypts with various intrinsic tensions. Quantitatively, mechanosensing lowered the differential tension threshold for bistability (that is, the boundary between bulged and budded phases). As a key consequence, we found that the tension asymmetry $\epsilon$, previously inferred from Myh9-GFP in budded crypts, is now above the theoretical threshold for bistability, enabling budded organoids to robustly remain budded on arbitrarily large lumen inflation (Figs. 3f and 4a,b and Supplementary Note 4.2). As an additional quantitative control, we also found that the threshold was still above the tension asymmetry $\epsilon$ from bulged crypts, supporting the experimental observation that bulged crypts require lumen volume decrease to bud (Figs. 3f and 4a,b).

## Quantitative morphogenetic trajectories of organoids

To summarize these findings, we derived the predicted trajectory of organoids with mechanosensing (Fig. 4a), which can reproduce the bistable hysteresis on both crypt morphology and crypt cellular tensions (Fig. 4b). Lumen shrinkage is critical during normal crypt morphogenesis, by both decreasing the cost associated with tissue bending (passive mechanical effect) and increasing the differential tension (active mechanosensing effect). By contrast, subsequent volume inflation in budded organoids does not cause the system to transition back to open-crypt morphologies and crypt cellular tensions are maintained. More quantitatively, the model can simultaneously fit cell tension data from both bulged and budded samples (Fig. 4c), with the single fitting parameter (intrinsic differential tension in the crypt) of *σ* = 0.02 (Supplementary Note 4.3).

Second, we sought to challenge the model with further temporal pharmacological perturbations (Extended Data Fig. 3c,d). We had previously shown that impairing contractility with blebbistatin in budded crypts results in crypt opening, that is, reversal towards a monostable bulged state^22^. However, the model also makes the key prediction that if we wash out blebbistatin but maintain the organoid in the swollen state, crypts should not be able to revert to budded morphologies (Fig. 4a), as the organoids go back to the bistable-parameter region from a budged state. To test this, we first treated budded organoids for 12 h with both blebbistatin and PGE, resulting in swollen organoids with bulged crypts (Extended Data Fig. 3d). We then washed out the blebbistatin, but retained the PGE. Strikingly, organoids remained swollen for around 12–24 h, and crypts did not revert to their budded morphology despite the blebbistatin washout (Extended Data Fig. 3d), consistent with the model. This provides further evidence that bistability confers history-dependent properties to crypt morphogenesis. Furthermore, after this time period, the lumen volume started decreasing in some organoids despite the presence of PGE, and we found that this was correlated to crypt budding, as that during normal organoid morphogenesis. This shows that pharmacological treatment did not have secondary effects that would cause an irreversible loss of budding ability (Extended Data Fig. 3d).

Finally, a non-trivial prediction from this model is that crypt morphologies should first vary relatively little with increasing apical tension and decreasing volume, before abruptly changing at the critical transition point. Thus, although crypt apical tension and lumen volume change gradually^22^, this would predict that the transition to a budded crypt should be fast as the organoid switches from one stable minimum of its energy landscape to another (snap-through bifurcation). Interestingly, plotting the crypt opening angles for different organoids as a function of time revealed a phase of abrupt closure (within 20 min; Fig. 4d), compared with a total time of around 24 h for full organoid morphogenesis (Fig. 1b and ref. ^22^).

## Discussion

In this work, we have combined a minimal description of a 3D epithelial monolayer shape, together with mechanosensitive couplings. Although we had previously shown that different cell fates pattern different mechanical properties^22^, here we further show that this provides a necessary but not sufficient condition for crypt budding, as additional feedback from enterocyte-mediated lumen volume changes modulate crypt tensions via mechanosensation. We constrain this theory based on detailed morphometric measurements of cell shape in intestinal organoids as well as live reporters of myosin activity on mechanical perturbations, namely, lumen inflation. We show that a bistable region in the organoid phase space generically arises, which can explain a number of experimental features on how lumen volume changes can mediate different responses at different morphogenetic time points (Figs. 1a and 4a). Indeed, the presence of bistability confers strong hysteretic behaviour to the system, so that lumen volume changes can be a key control parameter during the regular process of organoid morphogenesis as well as be rendered irrelevant once budded crypt morphogenesis is completed (Fig. 4e). This bears conceptual similarities to the role of prepatterned versus self-organized/self-reinforcing cues in other developmental systems, such as the mechanochemical interplay of embryo polarization in *Caenorhabditis elegans*^69,70^. This might, therefore, be a general mechanism that could ensure the robustness and irreversibility of morphogenesis in multicellular systems. The next important step would be to understand the molecular mechanosensing mechanisms that mediate the feedback from crypt morphology to actomyosin localization. Our inflation and deflation data suggest that this feedback is relatively fast (15–30 min) and reversible, although further work could also dissect whether additional feedback can occur on longer timescales of days, for instance, via cell fate maturation.

Our work also provides an example of the emerging role of fluid lumen pressure in controlling morphogenesis^11,14,17,19^. Compared with other types of biological force, fluid pressure has the advantage of being intrinsically long ranged. For instance, in intestinal morphogenesis, an outstanding question that remains is the nature of coordination between the specification and maturation of different crypts, both in vivo and in vitro. In organoids, lumen volume changes would be expected to provide a global cue that is felt equally among all the crypts of an organoid regardless of its size. Coupled with mechanosensing, this mechanism could allow for the synchronization of crypt morphogenesis, especially as the earlier crypt fate specification events are rather asynchronous^37^. Given that crypt fission was also shown to be dependent on lumen volume changes^38^, such mechanism could also act at multiple different time points during development. Importantly, even without fluid pressure, in vivo crypts can still be subjected to other external forces, arising, for instance, from the constriction of the smooth muscle layers^9^ or from the osmotic swelling of villus cells^22^. To test whether our proposed mechanism might also apply to geometries closed to the in vivo situation within an open tube, we performed additional theoretical analyses, in which crypts are under apical constriction and experience external forces directly from the surrounding villus tissue. Interestingly, we found a phase diagram very similar to the case of 3D organoids, showing that our mechanochemical bistability mechanism does not rely on a closed lumen, but generically arises in the presence of out-of-plane bending forces in the crypt combined with in-plane forces exerted by villus cells (Extended Data Fig. 10d,e and Supplementary Note 6). In the future, it will be important to understand—at the cellular and molecular levels—the mechanisms of the relationship between osmotic forces, lumen volume, crypt geometry and actomyosin accumulation, as well as to test whether similar principles of bistability and robustness of morphogenesis hold in other developmental and organoid systems.

## Methods

### Animal work

All the animal experiments were approved by the Basel Cantonal Veterinary Authorities and conducted in accordance with the Guide for the Care and Use of Laboratory Animals. Male and female outbred mice from 7 weeks old onwards were used for generating organoid lines of wild-type C57BL/6 and Lgr5-DTR-EGFP as reported previously^22^. One male mouse at 10 weeks was used to generate the organoid line of LifeAct-GFP. One male mouse at 10 weeks was used to generate the organoid line of Myh9-GFP.

Mouse lines used: wild-type C57BL/6 (Charles River Laboratories), Lgr5-DTR-EGFP (de Sauvage’s laboratory, Genentech), LifeAct-GFP (T. Hiiragi’s laboratory, EMBL) and Myh9-GFP (Lennon-Duménil’s laboratory, Institut Curie).

Mice were kept in housing conditions with 12 h light/12 h dark cycle, 18–23 °C ambient temperature and 40–60% humidity.

### Organoid culture

Organoids were generated from isolated crypts of the murine small intestine as previously described^22^. Organoids were kept in IntestiCult Organoid Growth Medium (STEMCELL Technologies) with 100 μg ml^–1^ penicillin–streptomycin for amplification and maintenance.

### Time-course experiments of fixed organoid samples

The method was adapted as described before^22^. Organoids were collected 5–7 days after passaging and digested with TrypLE (Thermo Fisher Scientific) for 20 min at 37 °C. Dissociated cells were passed through a cell strainer with a pore size of 30 μm (Sysmex). The collected cells were mixed with Matrigel (Corning) in an ENR medium to Matrigel ratio of 1:1(15). In each well of a 96-well plate, 5 μl droplets with 2,500 cells were seeded. After 15 min of solidification at 37 °C, 100 μl of the medium was overlaid. From day 0 to day 1, ENR was supplemented with 25% Wnt3a-conditioned medium (Wnt3a-CM), 10 μM Y-27632 (ROCK inhibitor, STEMCELL Technologies) and 3 μM of CHIR99021 (GSK3B inhibitor, STEMCELL Technologies, cat. no. 72054). From day 1 to day 3, ENR was supplemented with 25% Wnt3a-CM and 10 μM Y-27632. From day 3 to day 5, only ENR was added to the cells. Wnt3a-CM was produced in-house by Wnt3a L-cells (kind gift from Novartis).

### Compound treatments

Compound treatments were tested in a dilution series of various concentrations from 1 mM to 5 nM as that in a previous study^22^.

Single cells derived from LifeAct-GFP organoids were plated in a 96-well plate chamber and exposed to 0.5 μM PGE (kind gift from Novartis) or 0.5 µM dimethyl sulfoxide (Sigma-Aldrich, cat. no. D8418) diluted in an ENR medium, from 72 h for 24 h (dimethyl sulfoxide; Fig. 1b), from 96 h for 2 h (PGE, Fig. 1d) or from 72 h for 10 h (PGE; Fig. 1c).

Single cells derived from Myh9-GFP organoids were plated in an ibidi µ-Slide eight-plate chamber and cultured in a time-course medium till 84 h or 96 h for laser nanosurgery (Fig. 2e) or PGE treatment (Fig. 3c,d).

Single cells derived from Lgr5-DTR-GFP organoids were plated in a 96-well plate chamber and cultured in a time-course medium until fixation at 72 h, 84 h or 96 h (Extended Data Fig. 1a–e).

Single cells derived from organoids wild-type C57BL/6 were plated in a 96-well plate and treated with 0.5 μM PGE (kind gift from Novartis) or 0.5 μM dimethyl sulfoxide in an ENR medium, from 72 h until fixation at 74 h, 84 h or 96 h, or from 96 h until fixation at 98 h (Fig. 1e).

### Organoid immunostaining and imaging

The method was adapted from that described before^22^. Primary and secondary antibodies were diluted in a blocking buffer and applied. Cell nuclei were stained with 20 μg ml^–1^ DAPI (4′,6-diamidino-2-phenylindole, Invitrogen) in phosphate-buffered saline for 5 min at room temperature. Cells were stained with 1 μg ml^–1^ of Alexa Fluor 647 carboxylic acid succinimidyl ester (CellTrace, Invitrogen) in a carbonate buffer (1.95 ml of 0.5 M NaHCO_3_, 50 μl of 0.5 M Na_2_CO_3_, both from Sigma-Aldrich, in 8 ml of water for creating 10 ml of buffer).

High-throughput imaging was done with an automated spinning-disc microscope from Yokogawa (Cell Voyager 7000S), with an enhanced CSU-W1 spinning disc (microlens-enhanced dual Nipkow-disc confocal scanner), a ×40 (numerical aperture, 0.95) Olympus objective and a Neo scientific complementary metal–oxide–semiconductor camera (Andor, 2,560 × 2,160 pixels). For imaging, an intelligent imaging approach was used in the Yokogawa CV7000 (Search First module of Wako software 1.0) as described before^22^. Also, *z* planes spanning a range up to 90 μm and 2 or 3 μm *z* steps were acquired.

Confocal imaging of the fixed samples was performed using a Nikon Ti2-E Eclipse inverted microscope with a motorized stand along with a Yokogawa CSU-W1 dual camera (CAM1 SN: X-11424; CAM2 SN: 11736) T2 spinning-disc confocal scanning unit, CFI P-Fluor ×40 oil/1.4 objective and VisiView 4.4.0.9 software. Laser lines used are Toptica iBeam Smart 405/488/639 nm and Cobolt Jive 561 nm. Laser power and digital gain settings were unchanged within a given session to permit a direct comparison of expression levels among organoids stained in the same experiment. Image stacks were acquired with a slice thickness of 2 μm or less.

Confocal imaging of live Myh9-GFP samples for two time points before and after PGE inflation was performed using the same microscope and imaging settings as those used in the confocal imaging of the fixed samples. Before PGE treatment, one to five organoids from one well were quickly imaged within 10 min. Then, PGE was added into the culture medium of the same well and put back to 37 °C tissue culture. After 20–30 min, organoid lumens are sufficiently inflated. Imaging was done from the same organoids for the time point after inflation.

### Time-course image analysis

Organoid segmentation in the maximum intensity projections (MIPs) was adapted from another work^22^. For each acquired confocal *z*-stack field, MIPs were generated. All the MIP fields of a well were stitched together to obtain the MIP-well overviews for each channel. The high-resolution well overviews were used for organoid segmentation and feature extraction. From the segmented MIPs, we measure and calculate the features of each individual organoid.

### Light-sheet microscopy

Light-sheet microscopy was conducted using LS1 Live light-sheet microscope system (Viventis) or a similar customized microscope system as described before^22^. Sample mounting was performed as described previously^22^. For organoid imaging, LifeAct-GFP organoids were collected and digested with TrypLE (Thermo Fisher Scientific) for 20 min at 37 °C. GFP-positive cells were sorted by fluorescence-activated cell sorting and collected in a medium containing advanced DMEM/F-12 with 15 mM HEPES (STEMCELL Technologies) supplemented with 100 μg ml^–1^ penicillin–streptomycin, 1× GlutaMAX (Thermo Fisher Scientific), 1× B-27 (Thermo Fisher Scientific), 1× N-2 (Thermo Fisher Scientific), 1 mM *N*-acetylcysteine (Sigma), 500 ng ml^–1^ R-Spondin (kind gift from Novartis), 100 ng ml^–1^ Noggin (PeproTech) and 100 ng ml^–1^ murine EGF (R&D Systems). 2,000 cells were then embedded in a 5 μl drop of Matrigel/medium in a 50/50 ratio. Drops were placed in the imaging chamber and incubated for 20 min before being covered with 1 ml of the medium. For the first 3 days, the medium was supplemented with 20% Wnt3a-CM and 10 μM Y-27632 (ROCK inhibitor, STEMCELL Technologies). For the first day, in addition, 3 μM of CHIR99021 (STEMCELL Technologies) was supplemented. After more than 2 days of culturing in a cell culture incubator, the imaging chamber was transferred to the microscope kept at 37 °C and 5% CO_2_. Different organoids were selected as the starting positions and imaged every 10 min. A volume of 150–200 μm was acquired with a *z* spacing of 2 μm between slices. The medium was exchanged manually under the microscopy every half-day.

### Data analysis

Tissue opening angles *θ*_*i*_ (Fig. 2a shows the schematic, with *i* = c, v representing the crypt and villus tissues, respectively), which is related to the degree of crypt opening (defined as *θ*_c_/(π – *θ*_v_)), can be inferred from the coordinate information of the characteristic points along the midplane of the tissue, by approximating the tissue as a spherical cap. For each region (crypt or villus) in each sample, three points are extracted from the organoid image by using the multipoint tool of Fiji (version 2.9.0)^71^: two at the boundary (crypt/villus), denoted as (*x*_1_, *y*_1_) and (*x*_3_, *y*_3_), and one in the middle, denoted as (*x*_2_, *y*_2_) (Extended Data Fig. 2a shows the schematic). The opening angle can be calculated as

$$\theta =\uppi -\arccos \frac{\left({x}_{1}-x\right)\left({x}_{2}-x\right)+\left({y}_{1}-y\right)\left({y}_{2}-y\right)}{\sqrt{\left[{\left({x}_{1}-x\right)}^{2}+{\left({y}_{1}-y\right)}^{2}\right]\times \left[{\left({x}_{2}-x\right)}^{2}+{\left({y}_{2}-y\right)}^{2}\right]}},$$

which involves the coordinate of the centre of the spherical tissue: $x=\frac{B}{2A}$, $y=-\frac{C}{2A}$, *A* = *x*_1_(*y*_2_ – *y*_3_) + *x*_2_(*y*_3_ – *y*_1_) + *x*_3_(*y*_1_ – *y*_2_), $B=\left({x}_{1}^{2}+{y}_{1}^{2}\right)\left(\,{y}_{2}-{y}_{3}\right)+\left({x}_{2}^{2}+{y}_{2}^{2}\right)\left({y}_{3}-{y}_{1}\right)+\left({x}_{3}^{2}+{y}_{3}^{2}\right)(\,{y}_{1}-{y}_{2})$ and $C=\left({x}_{1}^{2}+{y}_{1}^{2}\right)$ $\left({x}_{2}-{x}_{3}\right)+\left({x}_{2}^{2}+{y}_{2}^{2}\right)\left({x}_{3}-{x}_{1}\right)+\left({x}_{3}^{2}+{y}_{3}^{2}\right)({x}_{1}-{x}_{2})$.

Morphometric parameters, including cell height *h*_*i*_, cell width *d*_*i*_ and tissue radius *R*_*i*_, were extracted from the images of intestinal organoids by using Fiji. Cell height (that is, epithelial thickness) *h*_*i*_ and cell width *d*_*i*_ of each region (crypt or villus) were measured by the line tool of Fiji in at least three random positions and determined as the average value of these measurements. Tissue radius *R*_*i*_ is defined as the average value of the apical and basal radii (Supplementary Note 1.1), and was quantified by the oval tool of Fiji.

Myh9-GFP intensity ratios in single cells were measured using Fiji (version 2.9.0) as described before^22^.

Lumen volume calculation of the light-sheet data was proceeded as done previously^22^.

### Lumen breakage by laser nanosurgery

The method was adapted from previous studies^22^. In brief, images are captured using an LSM710 scanning confocal microscope using the ZEN Black software (the only version). The microscope is equipped with an incubation chamber to maintain the sample at 37 °C and to provide 5% CO_2_. Organoids were embedded in Matrigel and cultured in ibidi eight-well plates. Cutting was performed using a wavelength of 850 nm with a Chameleon Ultra laser. All the cuttings were performed in the villus region of the organoid. The cutting region (Fig. 2e, yellow dashed lines) was set as a rectangle of 0.9 µm × 50 µm, and the activation time was calculated by the scan speed of 1.25 ms pixel^–1^. Organoids were imaged with a Plan-Neofluar ×40/0.9 Imm Korr Ph3 objective lens before and after cutting.

### Statistics and reproducibility

All the statistical analysis was performed using the Python library SciPy. All the box-plot elements show 25% (Q1, lower bounds), 50% (median, black lines within the boxes) and 75% (Q3, upper bounds) quartiles, and whiskers denote 1.5 times the interquartile range (maxima, Q3 + 1.5× (Q3 – Q1); minima, Q1 – 1.5× (Q3 – Q1)) with outliers (rhombuses). In compound time-course experiments, we assumed that a minimum of around 50 organoids on day 4 would be sufficient to recognize differences between control and perturbations based on historical experiments. The sample size was determined based on previous related studies in the field^37,72–74^.

### Reporting summary

Further information on research design is available in the Nature Portfolio Reporting Summary linked to this article.

## Online content

Any methods, additional references, Nature Portfolio reporting summaries, source data, extended data, supplementary information, acknowledgements, peer review information; details of author contributions and competing interests; and statements of data and code availability are available at 10.1038/s41567-025-02792-1.

## Supplementary information

Supplementary Information Supplementary Notes 1–6.

Reporting Summary

Supplementary Video 1 A full stack of an organoid expressing LifeAct-GFP is acquired every 20 min for 16 h 30 min from day 3 till budding. Left panel, single plane intersecting the middle of the organoid. Right panel, maximum *z* projection. Experiments were repeated with at least five independent recordings. Scale bar, 50 μm.

Supplementary Video 2 A full stack of a budded organoid expressing LifeAct-GFP is acquired every 30 min from day 3.5 (bulged organoid). Right panel, maximum *z* projection. Experiments were repeated with at least five independent recordings. Scale bar, 50 μm.

Supplementary Video 3 A full stack of an organoid expressing LifeAct-GFP is acquired every 3 min for around 2 h from day 4 budded organoid. Left panel, single plane intersecting the middle of the organoid. Right panel, maximum *z* projection. Experiments were repeated with at least five independent recordings. Scale bar, 50 μm.

Supplementary Video 4 A full stack of a bulged organoid expressing Myh9-GFP is acquired when the change in the organoid lumen volume was visually notable. The organoid was treated with 0.5 μM PGE, cultured and recorded for 1.5 h. Experiments were repeated at least three times. Scale bar, 50 μm.

## Source data

Source Data Fig. 1 Statistical source data.

Source Data Fig. 2 Statistical source data.

Source Data Fig. 3 Statistical source data.

Source Data Fig. 4 Statistical source data.

Source Data Extended Data Fig. 1 Statistical source data.

Source Data Extended Data Fig. 2 Statistical source data.

Source Data Extended Data Fig. 3 Statistical source data.

Source Data Extended Data Fig. 5 Statistical source data.

Source Data Extended Data Fig. 6 Statistical source data.

**Extended Data Fig. 8:**
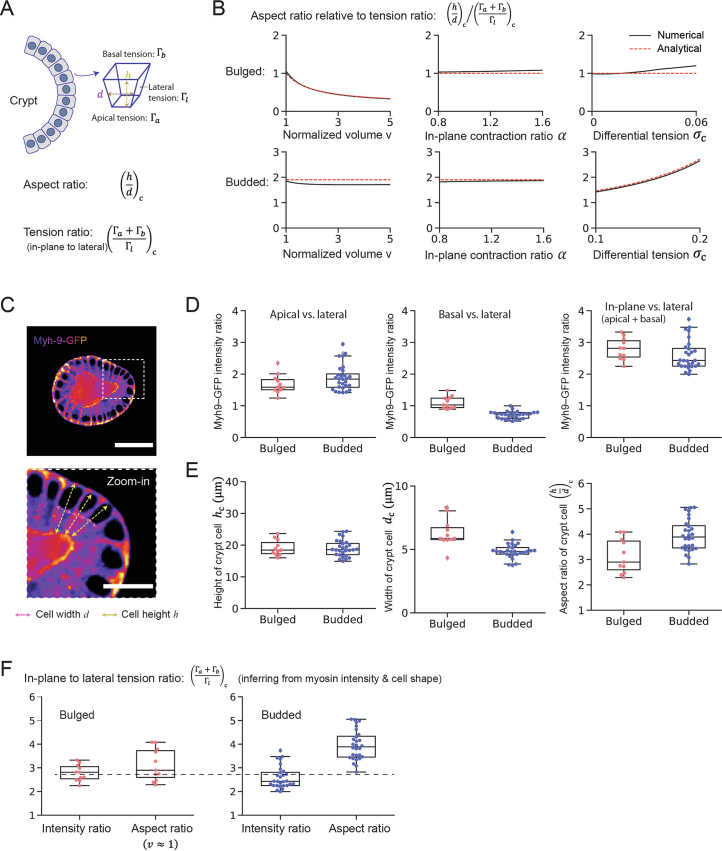
Link between mechanical and morphometric parameters. **A**. Definitions of crypt cellular aspect ratio ${(\frac{h}{d})}_{{\rm{c}}}$ and in-plane to lateral tension ratio ${(\frac{{\Gamma }_{a}+{\Gamma }_{b}}{{\Gamma }_{l}})}_{{\rm{c}}}$ (see SI Note section 3.1 for details). **B**. Sensitivity analysis for how model parameters affect the correlation between aspect ratio ${(\frac{h}{d})}_{{\rm{c}}}$ and in-plane to lateral tension ratio ${(\frac{{\Gamma }_{a}+{\Gamma }_{b}}{{\Gamma }_{l}})}_{{\rm{c}}}$ in both bulged and budded crypts: the dependence of the normalized value ${(\frac{h}{d})}_{{\rm{c}}}/{(\frac{{\Gamma }_{a}+{\Gamma }_{b}}{{\Gamma }_{l}})}_{{\rm{c}}}$ on normalized volume $v$ (left), in-plane contraction ratio $\alpha$ (middle) and differential tension ${\sigma }_{{\rm{c}}}$(right). We set $v=1,\,\alpha =1.2\,$ and ${\sigma }_{{\rm{c}}}=$ 0.03 (bulged) or 0.15 (budded) unless the parameter is varying. **C**. Representative organoid image with Myh-9-GFP staining and extraction of cellular height $h$ and width $d$. **D**. Quantification of Myh-9-GFP intensity ratio: apical, basal, and in-plane (sum of apical and basal) intensities are normalized by lateral intensity in each crypt (bulged: *N* = 11, budded: *N* = 28). **E**. Quantification of cellular height ${h}_{{\rm{c}}}$, width ${d}_{{\rm{c}}}$ and aspect ratio ${(\frac{h}{d})}_{{\rm{c}}}$ in both bulged and budded crypts (bulged: *N* = 11, budded: *N* = 28). Bulged samples are those after lumen breakage (that is $v\approx 1$, also shown in Fig. 2e), and in this state, cellular tension ratio and aspect ratio are expected to share the same value based on sensitive analysis B. **F**. Inference of in-plane to lateral tension ratio ${(\frac{{\Gamma }_{a}+{\Gamma }_{b}}{{\Gamma }_{l}})}_{{\rm{c}}}$ from corresponding Myh-9-GFP intensity ratio (that is right panel in D) and cellular aspect ratio (based on sensitive analysis B, tension ratio can also be inferred from aspect ratio in panel E). The box-plot elements show the 25% (Q1, lower bound), 50% (median, line within the box) and 75% (Q3, upper bound) quartiles, and the whiskers denote 1.5× the interquartile range (minimum = Q1–1.5$\times$(Q3–Q1), maximum = Q3$+$1.5$\times$(Q3–Q1)) with outliers (rhombuses). Scale bars: 50 µm (organoid) and 20 µm (magnification). Source data

Source Data Extended Data Fig. 8 Statistical source data.

**Extended Data Fig. 9:**
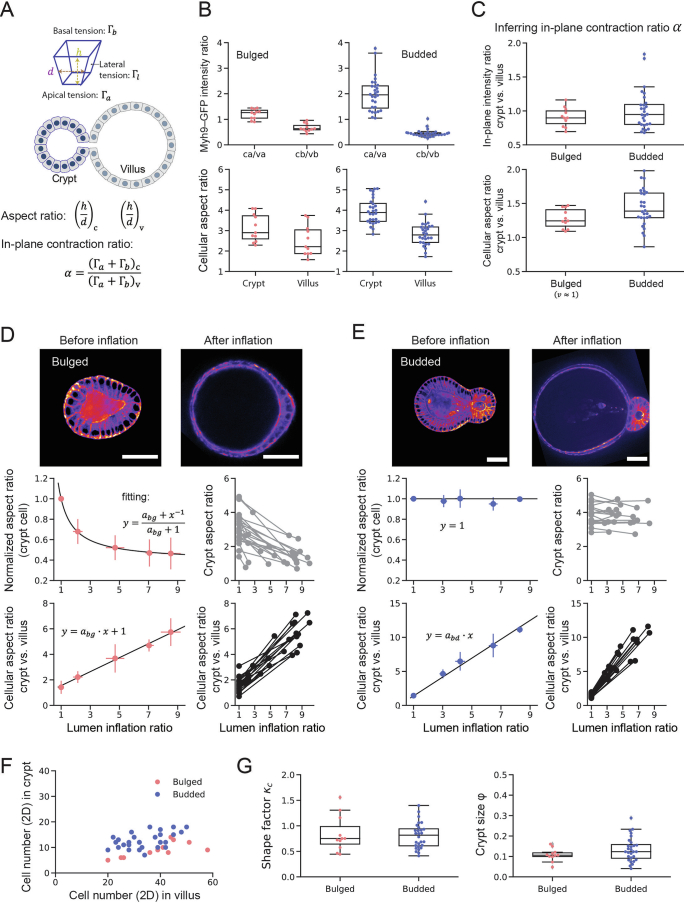
Quantification of mechanical parameters. **A**. Definitions of cellular aspect ratios ${(\frac{h}{d})}_{{\rm{c}}}$ and ${(\frac{h}{d})}_{{\rm{v}}}$, and in-plane contraction ratio $\alpha =\frac{{({\Gamma }_{a}+{\Gamma }_{b})}_{{\rm{c}}}}{{({\Gamma }_{a}+{\Gamma }_{b})}_{{\rm{v}}}}$, with subscripts c and v respectively denoting crypt and villus. **B**. Quantification of Myh-9-GFP intensity and cell shape: top, crypt to villus Myh-9-GFP intensity ratios, including crypt apical (ca) to villus apical (va), and crypt basal (cb) to villus basal (vb); bottom, quantification of cellular aspect ratios in crypt ${(\frac{h}{d})}_{{\rm{c}}}$ and that in villus ${(\frac{h}{d})}_{{\rm{v}}}$. **C**. Inference of in-plane contraction ratio $\alpha \,$ from both Myh-9-GFP intensity and cell shape: top, the ratio of in-plane Myh-9-GFP intensities (that is the sum of apical and basal intensities) of crypt to villus cell; bottom, the aspect ratio of crypt to villus cell ${(\frac{h}{d})}_{{\rm{c}}}/{(\frac{h}{d})}_{{\rm{v}}}$. In panel B and C, both bulged (*N* = 11) and budded (*N* = 28) samples are quantified. Bulged samples with lumen breakage (labelled as $v\approx 1$, also used in Fig. 2e) are chosen to quantify cellular aspect ratio as their cell shapes give direct inference of $\alpha$ (see SI Note section 3.2 for details). **D, E**. Fitting of the lumen inflation-induced morphometric variation with analytic formulae derived from the model. The images (top) show the organoid morphology and cell shape before and after lumen inflation by PGE treatment. Morphometric variation in cells is tracked by (middle) normalized aspect ratio of crypt cell ${(\frac{h}{d})}_{{\rm{c}}}^{{\rm{ifl}}}/{(\frac{h}{d})}_{{\rm{c}}}^{{\rm{ctr}}}$ (that is the value after lumen inflation ${(\frac{h}{d})}_{{\rm{c}}}^{{\rm{ifl}}}$ normalized by that before inflation ${(\frac{h}{d})}_{{\rm{c}}}^{{\rm{ctr}}}$, presented as mean ± SD, with data of ${(\frac{h}{d})}_{{\rm{c}}}^{{\rm{ctr}}}$ and ${(\frac{h}{d})}_{{\rm{c}}}^{{\rm{ifl}}}$ shown in the right panel), and (bottom) aspect ratio of crypt to villus cell ${(\frac{h}{d})}_{{\rm{c}}}/{(\frac{h}{d})}_{{\rm{v}}}$ (mean ± SD, with raw data shown in the right panel), in both bulged (panel D, *N* = 24) and budded (panel E, *N* = 19) organoids. **F**. Quantification of cell numbers in crypt and villus cross-sections (2D) from organoid images, which are used to estimate parameters in panel G (see SI Note section 3.3 and 4.3 for details). **G**. Measurement of shape factor ${\kappa }_{\tt{c}}$ (left) and crypt size $\varphi$ (right) in bulged (*N* = 11) and budded (*N* = 28) crypts. The box-plot elements show the 25% (Q1, lower bound), 50% (median, line within the box) and 75% (Q3, upper bound) quartiles, and the whiskers denote 1.5× the interquartile range (minimum = Q1–1.5$\times$(Q3–Q1), maximum = Q3$+$1.5$\times$(Q3–Q1)) with outliers (rhombuses). Scale bars: 50 µm. Source data

**Extended Data Fig. 10:**
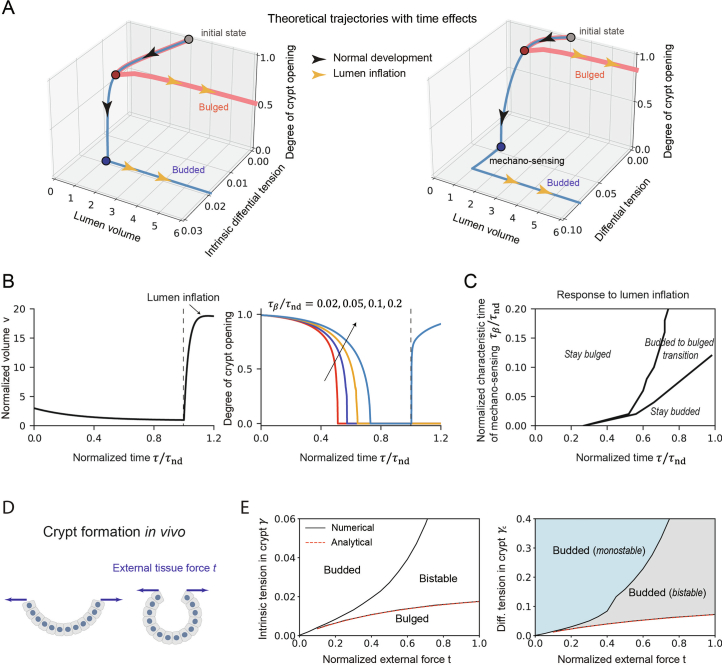
Crypt bistability mechanism: evaluation of time-dependent effects and extension to *in vivo* scenario. **A**. Theoretical trajectories showing the temporal evolution of crypt morphology, differential tension (that is intrinsic tension in the left panel, and total tension in the right one) and lumen volume, specified as normal morphogenesis (black arrows) and lumen inflation of both bulged and budded organoids (orange arrows). **B**. Temporal evolution of lumen volume in normal development and suing inflation treatment (left), and corresponding crypt morphologies with various time-scales of mechano-sensing (right). **C**. Morphological response to lumen inflation for crypts undergoing varying developmental time and showing varying mechano-sensing time-scales. Time-related parameters are normalized by ${\tau }_{{\rm{nd}}}$ (overall time of normal bulged-to-budded evolution). **D**. Extension of the model to the *in vivo* setting of an open geometry, where crypt budding can still be modulated and impeded by external force $t$ generated by other tissues (for example villus). **E**. Crypt morphology with curvature-sensation ($n=1$) in $\sigma -t$ diagram (left) and corresponding ${\sigma }_{{\rm{c}}}-t$ diagram showing the bistable crypt morphology (right).

Source Data Extended Data Fig. 9 Statistical source data.

## Data Availability

Source data are provided with this paper.

## Code availability

Codes used in the current study are from previous work^22^ and available on https://github.com/fmi-basel/glib-nature_cell_biology2021-materials.git.
