# Supplementary material for: Mechanochemical bistability of intestinal organoids enables robust morphogenesis
Source: Nat Phys. 2025 Feb 28;21(4):608–17. doi: 10.1038/s41567-025-02792-1 (PMC11999871; doi:10.1038/s41567-025-02792-1)
Supplement: Supplementary file 1 — Supplementary Notes 1–6. [file 41567_2025_2792_MOESM1_ESM.pdf]

nature physics

Supplementary information https://doi.org/10.1038/s41567-025-02792-1

Mechanochemical bistability of intestinal organoids enables robust morphogenesis

In the format provided by the authors and unedited

Supplementary Theory Note - Mechanochemical bistability of intestinal organoids enables robust morphogenesis

In this Supplementary note, we provide detailed analysis on morphological bistability of intestinal crypts. Crypt differential tension and lumen volume, two key parameters affecting crypt morphology, are treated independently at first to study how active cellular tensions and osmotic forces work synergistically to drive crypt morphogenesis, and the mechanical principle of morphological bistability that emerges in minimal 3D vertex descriptions of intestinal organoids. Then we consider a more realistic situation that includes coupling between these two driving forces, via mechano-sensation of crypt cells, and show how coupling differential tension with geometrical/mechanical cues gives rise to much wider regions of crypt bistability in the phase diagram. We further quantify mechanical parameters involved in the model, and validate the model by comparing theoretical predictions with experimental data.

1. Theory of crypt morphogenesis

In this section, we first analyzed the critical conditions for crypt shape bistability, based on a three-dimensional (3D) vertex model of crypt morphogenesis (described in further details in Yang et al. (2021)). By considering a scenario that decouples epithelial thickness and curvature, we show that one physical origin of bistability is linked to a purely mechanical feedback loop between epithelial thickness and bending moments, but that this purely physical bistability is only in a narrow range of parameters.

1.1. Two-region vertex model

An intestinal organoid is treated as a closed epithelial monolayer with two distinct regions, crypt and villus, encapsulating an incompressible fluid lumen. A two-region vertex model, with each region simplified as a homogeneous spherical cap, is developed to study the morpho-mechanics of organoids. In the following, we briefly introduce this 3D vertex model, more details can be found in Yang et al. (2021). We start from the free energy of a single cell. As mentioned in the main text, cell shape is regulated by a

balance of surface tensions Γ_a, Γ_b and Γ_l, where the subscripts a, b, and l respectively represent apical, basal, and lateral surfaces/domains. The apical and basal surfaces are simplified as squares with side lengths d_a and d_b, and the height of a cell is h.Then the free energy of a single cell is

$$f = \Gamma_a d_a^2 + \Gamma_b d_b^2 + \Gamma_l h(d_a + d_b). \tag{1}$$

Since each tissue region is a part of an idealized spherical epithelium with total cell number N', the side lengths are related to the region radii as $d_a = \sqrt{\frac{4\pi}{N'}}(R - \frac{h}{2})$, $d_b = \sqrt{\frac{4\pi}{N'}}(R + \frac{h}{2})$, with R the neutral radius of the epithelium (i.e. the average value of the radii of apical and basal surfaces). Then free energy (1) becomes

$$f = \frac{4\pi}{N'}[(\Gamma_a + \Gamma_b)R^2 + (\Gamma_b - \Gamma_a)Rh] + 2\sqrt{\frac{4\pi}{N'}} \cdot \Gamma_l Rh, \tag{2}$$

where $h \approx \frac{N' V_{\mathrm{e0}}}{4\pi R^2}$, with V_{e0} the cell volume independent from cell tensions.

The free energy of the whole organoid is the sum of the free energies of the two regions: $F = N_{\mathrm{c}} f_{\mathrm{c}} + N_{\mathrm{v}} f_{\mathrm{v}}$, where N_i and f_i are respectively cell number and cellular free energy in region i, with the index $i = \mathrm{c,v}$ denoting crypt and villus. The geometric variables (such as N_i, N_i', and R_i) involved in free energy F can be linked by the opening angle θ_i (sketched in Fig. S4A): (i) introduce an equivalent organoid radius R_{t} satisfying $V = 4\pi R_{\mathrm{t}}^3/3$, where V is the total organoid volume, then the region radius R_i can be related to radius R_{t} by considering volumetric relation $V = V_{\mathrm{c}} + V_{\mathrm{v}}$, with $V_i = \pi R_i^3(2 + 3\cos\theta_i - \cos^3\theta_i)/3$, and the geometric relation at the neck $R_{\mathrm{c}}\sin\theta_{\mathrm{c}} = R_{\mathrm{v}}\sin\theta_{\mathrm{v}}$. (ii) the ratio of cell number in crypt/villus (which is a spherical cap) to that in the whole spherical shell equals to the ratio of surface areas, that is $N_i/N_i' = (1 + \cos\theta_i)/2$. In the end, the free energy F is a function of two angles θ_{c} and θ_{v}, which quantify the degree of opening in crypt and villus, respectively.

Given that lateral tension Γ_l does not change significantly during development (Yang et al., 2021), we non-dimensionalize the free energy by introducing four dimensionless parameters:

- relative region size of the crypt $\varphi = \frac{N_{\mathrm{c}}}{N_{\mathrm{t}}}(N_{\mathrm{t}} = N_{\mathrm{c}} + N_{\mathrm{v}})$,

- in-plane tension ratio $\alpha = \frac{(\Gamma_a+\Gamma_b)_{\mathrm{c}}}{(\Gamma_a+\Gamma_b)_{\mathrm{v}}}$,
- apico-basal tension difference $\sigma_{\mathrm{c}} = \frac{1}{2}\left(\frac{\Gamma_a-\Gamma_b}{\Gamma_l}\right)_{\mathrm{c}}\sqrt{\frac{4\pi}{N_{\mathrm{t}}}}$, which related to the spontaneous curvature γ_{c} defined in Yang et al. (2021) as $\sigma_{\mathrm{c}} = -\gamma_{\mathrm{c}}$,
- normalized organoid volume $v = \left(R_{\mathrm{t}}/\tilde{R}_0\right)^3$, where $\tilde{R}_0$ is the radius of the spherical organoid in free state. Here, the "free state" means the tissue is free from any luminal fluid pressure and any tension difference in regions and surfaces.

The dimensionless free energy $\hat{F} = \frac{F}{\pi(\Gamma_a+\Gamma_b)_{\mathrm{v}}\tilde{R}_0^2}$ then reads

$$\hat{F} \approx v^{\frac{2}{3}}\left(\alpha G_{\mathrm{c}}^{-\frac{2}{3}} + G_{\mathrm{v}}^{-\frac{2}{3}}\right) + 16v^{-\frac{1}{3}}\left[\varphi^{\frac{3}{2}}G_{\mathrm{c}}^{\frac{1}{3}} + (1-\varphi)^{\frac{3}{2}}G_{\mathrm{v}}^{\frac{1}{3}} - \frac{1}{2}\varphi g_{\mathrm{c}}^{\frac{1}{3}}\sigma_{\mathrm{c}}\right], \tag{3}$$

where G_{c}, g_{c} and G_{v} are geometric parameters depend only on angles θ_{c} and θ_{v}. Possible morphologies (or mechanical equilibrium states) of organoids corresponds to the local minima of $\hat{F}$ (and corresponding θ_{c} and θ_{v}), which we calculated with the built-in function **FindMinimum** in Wolfram Mathematica.

Here we focus on crypt mechanics, and assume a constant cell volume for the whole organoid, without apical-basal tension difference in villus. In real circumstances, the cell volume in villus may changes due to active osmotic regulation, and elevated basal tension/myosin accumulation is observed in villus. However, our previous analysis (Yang et al., 2021) demonstrates that, cell swelling in villus only shows a volume effect similar to the lumen volume reduction and thus amplifies the impact of lumen shrinkage on the overall organoid volume, while the apical-basal tension difference in villus tissue has negligible influence on crypt morphologies. Therefore, our model can still capture the basic features of crypt morphogenesis.

1.2. Critical condition for bistability

Although the free energy (3) links system energy to crypt/villus shape (quantified by opening angles θ_{c} and θ_{v}), it remains hard to interpret qualitatively. However, considering crypts are usually much smaller than villi in intestinal organoids, we can

simplify the free energy of intestinal organoid as a function of crypt radius $\beta_c(= R_c/\tilde{R}_0)$ and opening angle of crypt θ_c (Yang et al., 2021):

$$\hat{F} \approx (\alpha s_c - \delta \sin^2 \theta_c)\beta_c^2 + \left(16\varphi^{\frac{3}{2}} s_c^{-\frac{1}{2}} - 8\varphi\sigma_c\right)\beta_c^{-1} - \frac{8}{3}\delta q_c \beta_c^3 v^{-\frac{1}{3}} + const., \qquad (4)$$

where $s_c = 2 + 2\cos\theta_c$, $q_c = \frac{1}{4}(2 + 3\cos\theta_c - \cos^3\theta_c)$, $\delta = 1 - (1-\varphi)^{\frac{3}{2}} v^{-1}$, and the last constant term only relevant to volume v.

The general criterion for a local minimum of free energy (4) reads

$$\frac{\partial \hat{F}}{\partial \beta_c} = 0, \qquad \frac{\partial \hat{F}}{\partial \theta_c} = 0,$$

$$H = \begin{vmatrix} \frac{\partial^2 \hat{F}}{\partial \beta_c^2} & \frac{\partial^2 \hat{F}}{\partial \beta_c \partial \theta_c} \\ \frac{\partial^2 \hat{F}}{\partial \theta_c \partial \beta_c} & \frac{\partial^2 \hat{F}}{\partial \theta_c^2} \end{vmatrix} > 0, \frac{\partial^2 \hat{F}}{\partial \beta_c^2} > 0, \frac{\partial^2 \hat{F}}{\partial \theta_c^2} > 0. \qquad (5)$$

A local minimum at $\theta_c \neq 0$ indicates an open crypt, while a local minimum at $\theta_c = 0$ means the crypt will be closed. A bistable state means the local energy minimum can locate at the boundary ($\theta_c = 0$) and in the region away from the boundary ($\theta_c \neq 0$) at the same time.

In a phase diagram as a function of diff. tension and lumen volume (e.g. Fig. 2A), the lower bound of bistable region corresponds to the occurrence of a local minimum at the boundary ($\theta_c = 0$), where $\left.\frac{\partial \hat{F}}{\partial \theta_c}\right|_{\theta_c=0} = 0$ and $\left.\frac{\partial^2 \hat{F}}{\partial \beta_c \partial \theta_c}\right|_{\theta_c=0} = 0$ always hold. Thus the critical condition reduces to: $\left.\frac{\partial \hat{F}}{\partial \beta_c}\right|_{\theta_c=0} = 0$ and $H|_{\theta_c=0} = 0$.We find $\frac{\partial \hat{F}}{\partial \beta_c} = 8\alpha\beta_c - 8\left(\varphi^{\frac{3}{2}} - \varphi\sigma_c\right)\beta_c^{-2} - 8\delta\beta_c^2\beta^{-1} = 0$ guarantees $\frac{\partial^2 \hat{F}}{\partial \beta_c^2} > 0$ and thus reduces the condition $H = 0$ as $\frac{\partial^2 \hat{F}}{\partial \theta_c^2} = 0$, which yields $\beta_c^3 = \varphi^{\frac{3}{2}}(\alpha + \delta)^{-1}$. Submitting this β_c into $\frac{\partial \hat{F}}{\partial \beta_c} = 0$, one can easily obtain the critical diff. tension as

$$\sigma_c = \varphi^{\frac{1}{2}} \frac{\delta}{\alpha + \delta}\left[1 + \frac{\varphi^{\frac{1}{2}}}{(\alpha+\delta)^{\frac{1}{3}} v^{\frac{1}{3}}}\right], \qquad \sigma_c \to \frac{\varphi^{\frac{1}{2}}}{\alpha + 1}\ (v \to \infty). \qquad (6)$$

The critical value is only weakly dependent on lumen volume (Fig. S4B) and approaches a finite value for large lumen volume.

The loss of bistability happens once the local minimum at $\theta_\mathrm{c} \neq 0$ disappears, which suggests the upper bound: $\frac{\partial \hat{F}}{\partial \theta_\mathrm{c}}\Big|_{\theta_\mathrm{c}\neq 0} = \frac{\partial \hat{F}}{\partial \beta_\mathrm{c}}\Big|_{\theta_\mathrm{c}\neq 0} = 0$ and $H|_{\theta_\mathrm{c}\neq 0} = 0$. The analytical solution is slightly more complicated. $\frac{\partial \hat{F}}{\partial \theta_\mathrm{c}} = 0$ requires $(\alpha + \delta\cos\theta_\mathrm{c})\beta_\mathrm{c}^2 - 8\varphi^{\frac{3}{2}} s_c^{-\frac{3}{2}}\beta_\mathrm{c}^{-1} - \delta \sin^2\theta_\mathrm{c}\beta_\mathrm{c}^3\beta^{-1} = 0$, while $\frac{\partial \hat{F}}{\partial \beta_\mathrm{c}} = 0$ leads to $(\alpha s_\mathrm{c} - \delta \sin^2\theta_\mathrm{c})\beta_\mathrm{c} - \left(8\varphi^{\frac{3}{2}} s_\mathrm{c}^{-\frac{1}{2}} - 4\varphi\sigma_\mathrm{c}\right)\beta_\mathrm{c}^{-2} - 4\delta q_\mathrm{c}\beta_\mathrm{c}^2\beta^{-1} = 0$. Considering crypts are much smaller than villi, we recognize $\beta_\mathrm{c}\beta^{-1}$ as a small quantity and find crypt radius β_c should satisfy $\beta_\mathrm{c}^3 \approx \frac{8\varphi^{\frac{3}{2}} s_\mathrm{c}^{-\frac{3}{2}}}{\alpha+\delta\cos\theta_\mathrm{c}} \approx \frac{8\varphi^{\frac{3}{2}} s_\mathrm{c}^{-\frac{1}{2}} - 4\varphi\sigma_\mathrm{c}}{\alpha s_\mathrm{c} - \delta\sin^2\theta_\mathrm{c}}$. This will greatly simplify the condition $H|_{\theta_\mathrm{c}\neq 0} = 0$ as a relation with only one unknown (θ_c): $[\delta + 3s_\mathrm{c}^{-1}(\alpha + \delta\cos\theta_\mathrm{c})](\alpha s_\mathrm{c} - \delta\sin^2\theta_\mathrm{c}) \approx 3(\alpha + \delta\cos\theta_\mathrm{c})^2$. It gives $\cos\theta_\mathrm{c} \approx \frac{\alpha}{\delta} - 2$, which further leads to $\beta_\mathrm{c} \approx 2^{\frac{1}{6}}\varphi^{\frac{1}{2}}\delta^{\frac{1}{2}}(\alpha - \delta)^{-\frac{5}{6}}$. One should notice that these solutions require $\alpha \geq \delta$. Submitting them into $\sigma_\mathrm{c} = \varphi^{\frac{1}{2}}\delta\left(\frac{1+\cos\theta_\mathrm{c}}{2}\right)^{\frac{1}{2}}\frac{1+\cos\theta_\mathrm{c}\beta_\mathrm{c}\beta^{-1}}{\alpha+\delta\cos\theta_\mathrm{c}-\delta\sin^2\theta_\mathrm{c}\beta_\mathrm{c}\beta^{-1}}$, which is obtained by $\frac{\partial \hat{F}}{\partial \theta_\mathrm{c}} = \frac{\partial \hat{F}}{\partial \beta_\mathrm{c}} = 0$, we can finally estimate the upper bound as $\sigma_\mathrm{c} \approx \frac{1}{2}\left(\frac{\varphi}{2}\frac{\delta}{\alpha-\delta}\right)^{\frac{1}{2}}\left[1 + 2^{-\frac{5}{6}}\left(\frac{\varphi}{\delta}\right)^{\frac{1}{2}}(\alpha-\delta)^{\frac{1}{6}}v^{-\frac{1}{3}}\right]$. We expect $\sigma_\mathrm{c} \to \frac{1}{2}\left(\frac{\varphi}{2}\frac{1}{\alpha-1}\right)^{\frac{1}{2}}$ for $v \to \infty$.

Importantly, these analytical lower and upper bounds are consistent with numerical solutions obtained from the full model, both in the $\sigma_\mathrm{c} - v$ and $\sigma_\mathrm{c} - \alpha$ phase diagrams (Fig. S4B).

1.3. One-dimensional energy landscape

In the above model, the full free energy (3) of an organoid depends on two parameters: opening angles θ_c and θ_v, while its simplified version (i.e. Eq. (4)) has normalized crypt radius β_c and crypt opening angle θ_c as independent parameters. As mentioned in criterion (5), equilibrium is only possible for $\frac{\partial \hat{F}}{\partial \beta_\mathrm{c}} = 0$ (and $\frac{\partial \hat{F}}{\partial \theta_\mathrm{c}} = 0$), which allows us to write β_c as a function of θ_c. Inputting the function $\beta_\mathrm{c}(\theta_\mathrm{c})$ into the free energy (4), we obtain $\hat{F} = const. + 12\varphi\alpha^{\frac{1}{3}}\Delta F(x)$, where $x = \cos\theta_\mathrm{c}$. $\Delta F(x)$ represents the mechanical energy related to crypt shape evolution, and is a one-dimensional (1D) energy landscape as a function of θ_c (Fig. S4C):

$$\Delta F(x) = \left[1 - \sigma_{\mathrm{c}}\left(\frac{1+x}{2\varphi}\right)^{\frac{1}{2}}\right]^{\frac{2}{3}}\left[1 - \frac{\delta}{2\alpha}(1-x)\right]^{\frac{1}{3}}[1 - \zeta(x)], \tag{7}$$

where $\zeta(x) \propto v^{-\frac{1}{3}}$ is a small quantity. For infinite volume ($v \to \infty$), the above equation reduces to Eq. (2) in the main text. Interestingly, differential tension has a much more drastic effect on the mechanical energy of closed/budded crypts compared to open/bulged one (Fig. S4F), as tissue bending increases the difference in apical to basal surface area. On the other hand, closed crypts are insensitive to lumen volume changes, as it is hardly deformed by luminal fluid pressure, while open crypt will deform with lumen volume (Fig. S4G).

In the perspective of system energy, the bistable-monostable transition is a result of the loss of energy barriers between bulged and budded states. In the bistable state (Fig. S4C), the energy landscape of an intestinal organoid includes two local minima (which shown as "valleys" in the landscape and respectively correspond to budded state at $\theta_{\mathrm{c}} = 0$ and bulged state with $\theta_{\mathrm{c}} > 0$) and one local maximum in between (i.e. the energy barrier, shown as a "peak" in the landscape). An increase in the differential tension σ_{c} would lower the system energies, where the local maximum and the budded-state energy decline much faster than the bulged-state energy (Fig. S4D). The bistable to monostable (budded) transition happens when the local maximum equals to the bulged-state energy and disappears (see the middle panel in Fig. S4E). On the other hand, increasing lumen volume v would lower the bulged-state energy and local maximum, but keep the budded-state energy unchanged (Fig. S4D). The equality of the local maximum to the budded-state energy indicates the loss of bistability (see the right panel in Fig. S4E).

These 1D landscapes can also help us better understand the phase diagram and trajectories of crypt morphogenesis. For example, increase differential tension until the system reaches the bistable state ($A \to C$ path in Fig. 2A and $\sigma_{\mathrm{c}} = 0.1 \to 0.15$ in Fig. S4F), where the landscape shows two "valleys". The crypt enters the nearest valley and keeps its bulged shape. To move to the other valley and get a closed shape, the crypt

would need external driving force to overcome the energy barrier between these two states/valleys.

The principle of crypt shape switched by deflation-inflation dynamics is also demonstrated in Fig. S4G. For a bulged organoid in an initially bistable state, volume deflation makes the bulged shape unfavorable and the crypt becomes monostable and moves towards the only energy minimum, which corresponds to the closed shape. Such bulged to budded shape transformation is validated by the lumen deflation experiment by osmotic shock (Fig. S3A, B). More quantitatively, we calculated the ratio of volume after vs. before osmotic shock (which is 0.42 ± 0.13, mean $\pm$ SD, see Fig. S3B). Based on the quantification in Fig. 2E, which suggests the bulged organoids have the normalized lumen volume $v \approx 2.77 \pm 1.27$, we can estimate the samples after osmotic shock have $v \approx 1.16$, just slightly above 1. The theory would predict (see Fig. 2A or 4B for instance) that such degree of lumen deflation is enough for the bulged to budded shape transformation, which is consistent with our experimental observations and the quantification of crypt opening degree.

The energy minimum in Fig. S4G still exists after the following volume inflation, as the closed crypt energy is insensitive to volume changes, and thus the crypt is "trapped" in this closed/budded shape.

1.4. Decoupling epithelial thickness and curvature

We attributed the bistability of crypt shape to the mechanical feedback between thickness and out-of-plane deformation of crypt epithelium: crypt bending drives epithelial thickening, which in turn promotes crypt bending by increasing "active bending moment" $M \sim (\Gamma_a - \Gamma_b)h$. To test this, here we set the active bending moment in the crypt to be independent on thickness by setting the thickness in this term constant (equal to thickness in deformation-free state: $\tilde{h} = V_{e0}/\tilde{d}^2$, with $\tilde{d} = \left(\frac{\Gamma_l V_{e0}}{\Gamma_a + \Gamma_b}\right)^{\frac{1}{3}}$ the corresponding cell width). Then the free energy of a single (crypt) cell is

$$f=\frac{4\pi}{N'}(\Gamma_a+\Gamma_b)R^2+\frac{4\pi}{N'}(\Gamma_b-\Gamma_a)R\tilde{h}+2\sqrt{\frac{4\pi}{N'}}\cdot\Gamma_l Rh. \qquad (8)$$

Note that we still allow other terms such as the one related to lateral tension (i.e. $\sim\Gamma_l Rh$ term), which contributes to the "bending resistance" of an epithelium, to depend on thickness (i.e. $h=\frac{N'V_{\mathrm{e0}}}{4\pi R^2}$ still holds and thickness still changes with out-of-plane deformation). The corresponding organoid free energy yields

$$\hat{F}\approx v^{\frac{2}{3}}\left(\alpha G_{\mathrm{c}}^{-\frac{2}{3}}+G_{\mathrm{v}}^{-\frac{2}{3}}\right)+16v^{-\frac{1}{3}}\left[\varphi^{\frac{3}{2}}G_{\mathrm{c}}^{\frac{1}{3}}+(1-\varphi)^{\frac{3}{2}}G_{\mathrm{v}}^{\frac{1}{3}}\right]-2v^{\frac{1}{3}}s_c g_{\mathrm{c}}^{-\frac{1}{3}}\sigma_{\mathrm{c}}. \qquad (9)$$

Interestingly, numerical results from analyzing the free energy (9) indicate bistable states no longer exists, validating our hypothesis that the coupling between thickness and out-of-plane deformation is responsible for bistability. Similar to Subsection 1.2, for this modified model, we can also give some analytic arguments on the search for bistable state. Free energy (9) can be approximated as

$$\hat{F}\approx(\alpha s_{\mathrm{c}}-\delta\sin^2\theta_{\mathrm{c}})\beta_{\mathrm{c}}^2+16\varphi^{\frac{3}{2}}s_{\mathrm{c}}^{-\frac{1}{2}}\beta_{\mathrm{c}}^{-1}-2\alpha^{\frac{2}{3}}\sigma_{\mathrm{c}}s_{\mathrm{c}}\beta_{\mathrm{c}}-\frac{8}{3}\delta q_{\mathrm{c}}\beta^3 v^{-\frac{1}{3}}+const.. \qquad (10)$$

Without loss of generality, we consider $v\to\infty$ in the following.

Again, the local energy minimum at $\theta_{\mathrm{c}}=0$ requires $\frac{\partial\hat{F}}{\partial\beta_{\mathrm{c}}}=0$ and $H=\frac{\partial^2\hat{F}}{\partial\beta_{\mathrm{c}}^2}\cdot\frac{\partial^2\hat{F}}{\partial\theta_{\mathrm{c}}^2}>0$. Considering $\frac{\partial^2\hat{F}}{\partial\theta_{\mathrm{c}}^2}=0$ ($\frac{\partial^2\hat{F}}{\partial\beta_{\mathrm{c}}^2}$, the other component in H, stays positive once $\frac{\partial\hat{F}}{\partial\beta_{\mathrm{c}}}=0$ is satisfied), we get the critical diff. tension for crypt closure: $\sigma_{\mathrm{c}}=\varphi^{\frac{1}{2}}\alpha^{-\frac{2}{3}}(\alpha-1)^{-\frac{1}{3}}$. For $\theta\neq 0$, $H>0$ (and $\frac{\partial^2\hat{F}}{\partial\beta_{\mathrm{c}}^2}>0$) always hold, and the two first-order partial derivatives in criterion (5) lead to the $\sigma_{\mathrm{c}}\sim\theta_{\mathrm{c}}$ relation: $\sigma_{\mathrm{c}}=\varphi^{\frac{1}{2}}\alpha^{-\frac{2}{3}}(\alpha-1)^{-\frac{1}{3}}\left(\frac{1+\cos\theta_{\mathrm{c}}}{2}\right)^{\frac{1}{2}}(\theta_{\mathrm{c}}\neq 0)$, which clearly shows the degree of crypt opening monotonously declines with the increase in differential tension. Importantly, this relation also implies the crypt should close at $\sigma_{\mathrm{c}}=\varphi^{\frac{1}{2}}\alpha^{-\frac{2}{3}}(\alpha-1)^{-\frac{1}{3}}$, the same threshold obtained by directly analyzing the scenario $\theta_{\mathrm{c}}=0$. In other words, once the differential tension reaches this threshold and the closed shape starts to emerge, open shapes are no longer possible for crypts.

Based on the above thickness-curvature coupling mechanism, one could also predict the shape transformation from open to closed morphological states would be harder for a crypt with unchanged thickness than the one thickening with bending. This is verified by the larger spontaneous curvature σ_{c} required for crypt budding (corresponds to open/closed boundary in phase diagrams) in Fig. S4H, compared to the full model (Fig. S4B).

2. Impacts of mechano-sensation on bistability

As mentioned in the main text, based on a number of experimental evidence, we hypothesized two possible mechano-sensation mechanisms, which respectively involve the influence of geometrical and mechanical cues on crypt differential tension. These two mechano-sensation modes are related to two basic deformation modes of cells: out-of-plane bending and in-plane stretching/compression. These deformations will change the radius of curvature R and cellular width (or side length) d, which can be revealed by writing the free energy (1) as:

$$f \approx (\Gamma_a + \Gamma_b)d^2 + 2\Gamma_l \frac{V_{\mathrm{e0}}}{d} + (\Gamma_b - \Gamma_a)\frac{V_{\mathrm{e0}}}{R}. \tag{11}$$

As a complete argument, in the following, we will sequentially discuss the $\sigma_{\mathrm{c}} - R$ and $\sigma_{\mathrm{c}} - d$ couplings, and respectively evaluate their impacts on crypt bistability by analyzing one-dimensional energy landscape and the lower bound for bistability in the diff. tension-volume diagram. We show that this drastically enhances the region of bistability in the phase diagram, which will be required to explain our data.

2.1. Curvature-sensation

In the curvature-sensing mechanism, an increase in crypt radius will reduce the apical to basal tension difference, which we parametrize by the relationship (Fig. S5A):

$$\sigma_{\mathrm{c}} = \sigma\left(\frac{R_{\mathrm{c}}}{\tilde{R}_0}\right)^{-n} = \sigma\beta_{\mathrm{c}}^{-n}, \tag{12}$$

where n is to evaluate the sensitivity of diff. tension to epithelial curvature/crypt radius R_{c} (away from its preferred state $\tilde{R}_0$ with fate-dependent intrinsic tension σ).

The above coupling makes it hard to get an analytical form of ΔF as in Eq. (7). Instead, we can numerically obtain this mechanical energy. Based on free energy (4), we still use equilibrium condition $\frac{\partial \hat{F}}{\partial \beta_c} = 0$ to get β_c as a function of θ_c, then injecting β_c into $\hat{F}$. Then total free energy $\hat{F}$ and ΔF, which are related by $\hat{F} = const. + 12\varphi\alpha^{\frac{1}{3}}\Delta F(x)$ (see Subsection 1.3), are only dependent on crypt opening angle θ_c. With increasing sensitivity n, the 1D energy decreases in the region with crypts closed/budded ($\theta_c = 0$) until a critical value where $\theta_c = 0$ becomes a local minimum (Fig. S5A), indicating curvature-sensation favor the closure of crypts.

The critical condition for crypt bistability with curvature-sensation can also be obtained by analyzing local energy minimum (Eq. (5)). Same with the discussion in Subsection 1.2, we rely on $\frac{\partial \hat{F}}{\partial \beta_c} = 0$ and $\frac{\partial^2 \hat{F}}{\partial \theta_c^2} = 0$ at $\theta_c = 0$ to get the lower bound, which suggests $\bar{\sigma}_c = \varphi^{\frac{1}{2}} \frac{\delta}{\alpha + \delta}(1 + \beta_c \beta^{-1})$, with $\bar{\sigma}_c = \sigma_c - \frac{d\sigma_c}{d\beta_c}\beta_c$ and $\beta_c = \varphi^{\frac{1}{2}}(\alpha + \delta)^{-\frac{1}{3}}$. This critical condition is similar to the scenario without mechano-sensation (i.e. $\bar{\sigma}_c$ equals to the differential tension in Eq. (6)), only now the left side includes an additional term $-\frac{d\sigma_c}{d\beta_c}\beta_c$. Finally, we have the critical diff. tension

$$\sigma_c|_{\theta_c=0} = (1+n)^{-1}\bar{\sigma}_c\,, \qquad (13)$$

and corresponding intrinsic value $\sigma = (1+n)^{-1}\left(\frac{\alpha}{\alpha+\delta}\right)^{\frac{n}{3}}\bar{\sigma}_c$ for curvature-sensation (see phase diagram Fig. S5C-D). The lower bound of bistability decreases with increasing sensitivity by a simple factor $(1+n)^{-1}$, further validating curvature-sensation enhances crypt bistability.

2.2. Force-sensation

We now introduce the cellular stretch ratio $\lambda_c = d_c/\tilde{d}_c$ as a morphometric parameter to evaluate the stretching degree of crypt cells, with $\tilde{d}_c = \left(\frac{\Gamma_l V_{e0}}{\Gamma_a + \Gamma_b}\right)_c^{\frac{1}{3}}$ the cell width in force-free state (see schematic Fig. S6A). Under a force-sensing mechanism, to parametrize crypt differential tension as a function of crypt stretching, we can use

the following relationship:

$$\sigma_{\mathrm{c}} = \sigma \left(\frac{d_{\mathrm{c}}}{\tilde{d}_{\mathrm{c}}} \right)^{-n} = \sigma \lambda_{\mathrm{c}}^{-n} \,. \tag{14}$$

At the tissue scale, stretch ratio can be written as $\lambda_{\mathrm{c}} = \beta_{\mathrm{c}}/\tilde{\beta}_{\mathrm{c}}$, with $\tilde{\beta}_{\mathrm{c}} = 2\varphi^{\frac{1}{2}}\alpha^{-\frac{1}{3}}s_{\mathrm{c}}^{-\frac{1}{2}}$ the reference radius of a crypt at zero stretch/compression. Note that crypt stretching is related to crypt radius, meaning that force and curvature sensing could be hard to distinguish experimentally.

We can also consider the hypothesis that crypt cells directly sense stretching forces instead of deformation, in particular the local epithelial tension T_{c} (in-plane force per unit cell width engendered by neighboring cells). Then the total in-plane force is $T_{\mathrm{c}} \cdot d_{\mathrm{c}} = \frac{1}{2}\frac{\partial f_{\mathrm{c}}}{\partial d_{\mathrm{c}}}$, where pre-factor 1/2 arises from isotropic deformation. Recalling the free energy (11), we find the force-deformation relation follows a simple form: normalized force $t_{\mathrm{c}} = \frac{T_{\mathrm{c}}}{(\Gamma_a+\Gamma_b)_{\mathrm{c}}} = 1 - \lambda_{\mathrm{c}}^{-3}$. To avoid a singularity, we can consider the dependency $\sigma_{\mathrm{c}} = \sigma(1-t_{\mathrm{c}})^m$, which leads to the same form as the $\sigma_{\mathrm{c}} - \lambda_{\mathrm{c}}$ coupling described above (for $m = n/3$).

Unlike in the case of curvature-sensation, the differential tension is a function of both crypt radius and opening angle: $\sigma_{\mathrm{c}}(\beta_{\mathrm{c}}, \theta_{\mathrm{c}})$. 1D energy landscapes in Fig. S6A show that this force-sensation can also generically endow a crypt with bistability. Based on the same critical condition: $\frac{\partial \hat{F}}{\partial \beta_{\mathrm{c}}} = 0$ and $\frac{\partial^2 \hat{F}}{\partial \theta_{\mathrm{c}}^2} = 0$ at $\theta_{\mathrm{c}} = 0$, we obtain the lower bound (Fig. S6B):

$$\sigma_{\mathrm{c}}|_{\theta_{\mathrm{c}}=0} \approx \frac{\varphi^{\frac{1}{2}}\delta}{\alpha+(1+n)\delta}\left[1+\frac{\varphi^{\frac{1}{2}}(\alpha+\delta)}{(\alpha+(1+n)\delta)^{\frac{4}{3}}v^{\frac{1}{3}}}\right], \quad \sigma_{\mathrm{c}} \to \frac{\varphi^{\frac{1}{2}}}{\alpha+1+n}\ (v\to\infty)\,. \tag{15}$$

This critical value decreases with increasing force-sensitivity, indicating force-sensation also enhances crypt bistability.

2.3. Double feedback loops

In summary, there are two types of feedbacks related to crypt bistability (Fig. S5E): Feedback 1 is the mechanical coupling between epithelial thickness and curvature

(arising without any type of mechano-sensation), Feedback 2 is the coupling between crypt apico-basal tension difference and tissue deformation (with mechano-sensation). However, the above discussions on the impacts of mechano-sensation/Feedback 2 (see Subsection 2.1 and 2.2) are based on the model that intrinsically includes Feedback 1. To check if mechano-sensation alone can lead to bistability, we also used the modified free energy (9), which has removed the influence of Feedback 1, to analyze the lower bound for bistability. The result shows mechano-sensation can still help the crypt to enter bistable states, although the sensitivity factor n then needs to reach a threshold value (Fig. S5F). These two feedbacks thus work synergistically to enhance crypt bistability.

3. Quantification of mechanical parameters

The quantification of the active cellular tensions is typically a challenging point in the field. Here, we consider two types of easily accessible metrics related to cellular tensions: i) Myosin intensity, which has been found to strongly correlate with cellular tension not only in intestinal organoids (Yang et al., 2021) but also in a range of tissues and developmental settings (Streichan et al., 2018); ii) cellular morphometric parameters, which are speculated to be quantitatively related to cellular active tension, given that cellular tensions control cell shapes. In this Section, we tested whether either Myosin intensities or morphometric parameters could be used to estimate the tension-related parameters in the model. Importantly, we found that both methods were quite consistent to estimate tension-related parameters, including in-plane to lateral tension ratio $(\Gamma_a + \Gamma_b)/\Gamma_l$ and in-plane contraction ratio α. Such consistency provides a key check on our quantifications. We can also infer the differential tension σ_c, an important parameter in the model, from Myosin intensity ratio of apical to basal surfaces. Our quantification strategy is summarized in a Table (see Fig. S7).

3.1. Link between mechanical and morphometric parameters

Considering the fact that cellular tensions control cell shapes, we can use cellular

morphometric parameter to constrain the values of specific tension ratios, and then compare it to the values from Myosin intensity ratios. For example, we find that the cellular aspect ratio (i.e. cell height to width ratio $\frac{h}{d}$), an easily accessible morphometric parameter, is mainly controlled by the in-plane to lateral tension ratio $\frac{\Gamma_a+\Gamma_b}{\Gamma_l}$ (see Fig. S8A for schematic).

In the general form, the aspect ratio of crypt cells yields $\left(\frac{h}{d}\right)_{\mathrm{c}} = \frac{\tilde{h}_0}{\tilde{d}_0}\left(2\varphi^{\frac{1}{2}}\right)^3\left(s_{\mathrm{c}}^{\frac{1}{2}}\beta_{\mathrm{c}}\right)^{-3}$, with $\tilde{h}_0$ and $\tilde{d}_0$ respectively the free-state cell height and width. In the free state, the cellular aspect ratio is solely affected by (villus) in-plane to lateral tension ratio: $\frac{\tilde{h}_0}{\tilde{d}_0} = \left(\frac{\Gamma_a+\Gamma_b}{\Gamma_l}\right)_{\mathrm{v}}$. For a bulged crypt, $\theta_* = \pi - \theta_{\mathrm{c}}$ is a small angle, and we have $s_{\mathrm{c}}^{\frac{1}{2}}\beta_{\mathrm{c}} \approx \bar{\theta}_* = \theta_*\beta_{\mathrm{c}}$, so that the free energy (4) simplifies as $\hat{F} \approx (\alpha-\delta)\bar{\theta}_*^2 + 16\varphi^{\frac{3}{2}}\bar{\theta}_*^{-1} - 8\varphi\sigma_{\mathrm{c}}\beta_{\mathrm{c}}^{-1} + const.$. The mechanical equilibrium condition $\frac{\partial\hat{F}}{\partial\bar{\theta}_*} = 0$ leads to $\bar{\theta}_* = 2\varphi^{\frac{1}{2}}(\alpha-\delta)^{-\frac{1}{3}}$, which gives us the cellular aspect ratio in bulged crypts as:

$$\left(\frac{h}{d}\right)_{\mathrm{c}} = \left(\frac{\Gamma_a+\Gamma_b}{\Gamma_l}\right)_{\mathrm{c}}\left(1-\frac{1-v^{-1}}{\alpha}\right). \qquad (16)$$

Sensitivity analysis based on numerical results of the full free energy (3) and analytical expression (16) (Fig. S8B) shows that cellular aspect ratio in bulged crypts decreases with lumen inflation, but is only set by tension ratio $\left(\frac{\Gamma_a+\Gamma_b}{\Gamma_l}\right)_{\mathrm{c}}$ close to $v=1$ (insensitive to other tension-related parameters). Thus, the cellular aspect ratio of samples with lumen breakage (i.e. those in Fig. 2E), which mimics the scenario with $v \approx 1$, can be used to estimate $\left(\frac{\Gamma_a+\Gamma_b}{\Gamma_l}\right)_{\mathrm{c}}$ (Fig. S8C and S8E).

A budded crypt is defined by $\theta_{\mathrm{c}} \approx 0$, and its radius can be obtained by considering mechanical equilibrium $\frac{\partial\hat{F}}{\partial\beta_{\mathrm{c}}} = 0$. For both $\sigma_{\mathrm{c}} - R_{\mathrm{c}}$ and $\sigma_{\mathrm{c}} - d_{\mathrm{c}}$ couplings, one gets the same result: $s_{\mathrm{c}}^{\frac{1}{2}}\beta_{\mathrm{c}} = 2\varphi^{\frac{1}{2}}\alpha^{-\frac{1}{3}}\left[1-(1+n)\varphi^{-\frac{1}{2}}\sigma_{\mathrm{c}}\right]^{\frac{1}{3}}$, with n the sensitivity factor, and corresponding aspect ratio

$$\left(\frac{h}{d}\right)_{\mathrm{c}} = \left(\frac{\Gamma_a+\Gamma_b}{\Gamma_l}\right)_{\mathrm{c}}\left[1-(1+n)\varphi^{-\frac{1}{2}}\sigma_{\mathrm{c}}\right]^{-1}. \qquad (17)$$

Besides the in-plane to lateral tension ratio, the cell shape in budded crypts is now also

dependent on differential tension σ_c (Fig. S8B). For a closed epithelium, higher apical tension constricts the epithelium inward, leading to a decrease in tissue radius /cell width and an increase in tissue thickness/cell height. After removing the effects of differential tension σ_c and sensitivity factor n (respectively estimated in Subsection 3.3 and 4.1), we can estimate the in-plane to lateral tension ratio in budded crypts from Eq. (17).

For this, we computed $\left(\frac{\Gamma_a+\Gamma_b}{\Gamma_l}\right)_c$ from the Myh-9-GFP intensity ratio in the apical, lateral and basal surfaces (Fig. S8C), for both bulged and budded crypts (Fig. S8D, median values). Interestingly, we found that the obtained values were quite close to the values inferred from morphometrics (Fig. S8F): $\left(\frac{\Gamma_a+\Gamma_b}{\Gamma_l}\right)_c = 2.8$ (intensity ratio) vs. 2.9 (aspect ratio) for bulged crypts, and 2.4 (intensity ratio) vs. 1.9 (estimated from aspect ratio 3.9) for budded ones. Such consistency provides evidence that Myosin intensities can serve as reliable data for the evaluation of cellular tension.

3.2. Quantification of in-plane contraction ratio α

As an important mechanical parameter affecting organoid morphology, the in-plane contraction ratio $\alpha = \frac{(\Gamma_a+\Gamma_b)_c}{(\Gamma_a+\Gamma_b)_v}$ compares the in-plane tensions in crypt to villus (Fig. S9A). For both crypt and villus cells, we can respectively obtain their Myosin intensities in apical and basal cell surfaces, and calculate the crypt to villus intensity ratio (top panel in Fig. S9B-C), which suggests α to be close to 1 for both bulged and budded samples (top panel in Fig. S9C).

The in-plane contraction ratio can also be inferred by comparing the cellular aspect ratio in crypt and villus. The analytical aspect ratio of crypt cells has been obtained in Subsection 3.1, and the aspect ratio of villus cells yields $\left(\frac{h}{d}\right)_v = \left(\frac{\Gamma_a+\Gamma_b}{\Gamma_l}\right)_v \cdot v^{-1}$ for both bulged and budded organoids. Then we have the dependence of ratio $\left(\frac{h}{d}\right)_c / \left(\frac{h}{d}\right)_v$ on tension ratio α as

$$\left(\frac{h}{d}\right)_{\mathrm{c}} \Big/ \left(\frac{h}{d}\right)_{\mathrm{v}} = (\alpha - 1)v + 1 \qquad \text{(bulged)},$$
$$\left(\frac{h}{d}\right)_{\mathrm{c}} \Big/ \left(\frac{h}{d}\right)_{\mathrm{v}} = \alpha \left[1 - (1+n)\varphi^{-\frac{1}{2}}\sigma_{\mathrm{c}}\right]^{-1} v \qquad \text{(budded)}. \tag{18}$$

We quantified bulged samples with lumen breakage ($v \approx 1$, see Fig. 2E) in the bottom panels of Fig. S9B-C. Consistent with the Myosin intensity data, our measurement results of $\left(\frac{h}{d}\right)_{\mathrm{c}} / \left(\frac{h}{d}\right)_{\mathrm{v}}$ are close to 1, although in general crypt cells have slightly larger aspect ratios than villus cells (Fig. S9C). With the analytical expression (18), this constrains the ratio α of bulged samples to be slightly above 1, consistent with our previous fitting results of thickness ratio $h_{\mathrm{c}}/h_{\mathrm{v}}$ (Yang et al., 2021). Unlike thickness h, cellular aspect ratio h/d is independent on cellular volume, thus $\left(\frac{h}{d}\right)_{\mathrm{c}} / \left(\frac{h}{d}\right)_{\mathrm{v}}$ would not be affected by possible volume difference in crypt vs. villus cells, which becomes non-negligible in budded organoids (Yang et al., 2021).

Direct fitting of the variation of cellular aspect ratios upon lumen inflation provides additional information on in-plane contraction ratio α (Fig. S9D-E). First, we consider the variation of crypt cell aspect ratio $\left(\frac{h}{d}\right)_{\mathrm{c}}$ with lumen inflation (middle panels in Fig. S9D-E). For each sample, we use the aspect ratio in its normal (i.e. control) state to normalize the value in the inflated state, that is $\left(\frac{h}{d}\right)_{\mathrm{c}}^{\mathrm{ifl}} / \left(\frac{h}{d}\right)_{\mathrm{c}}^{\mathrm{ctr}}$, and employ the same normalization procedure to get the lumen inflation ratio $\bar{v}$. The corresponding analytical relation used for the fitting can be obtained by Eqs. (16) and (17):

$$\left(\frac{h}{d}\right)_{\mathrm{c}}^{\mathrm{ifl}} \Big/ \left(\frac{h}{d}\right)_{\mathrm{c}}^{\mathrm{ctr}} = \left(\frac{a_{\mathrm{bg}} + \bar{v}^{-1}}{a_{\mathrm{bg}} + 1}\right) \qquad \text{(bulged)},$$
$$\left(\frac{h}{d}\right)_{\mathrm{c}}^{\mathrm{ifl}} \Big/ \left(\frac{h}{d}\right)_{\mathrm{c}}^{\mathrm{ctr}} = 1 \qquad \text{(budded)}, \tag{19}$$

with fitting parameter $a_{\mathrm{bg}} = (\alpha - 1)v_0$, and $v_0 = v/\bar{v}$ the volume of a sample in its normal state. The fitting of bulged samples suggests $a_{\mathrm{bg}} = 0.64$ (Fig. S9D), and data of budded samples also indicate cellular aspect ratio is unchanged with lumen inflation (Fig. S9E), consistent with the analytical result (19). Second, we consider the variation of $\left(\frac{h}{d}\right)_{\mathrm{c}} / \left(\frac{h}{d}\right)_{\mathrm{v}}$ with lumen inflation (bottom panels in Fig. S9D-E), and obtain the

predicted dependency by rewriting Eq. (18) as

$$\left(\frac{h}{d}\right)_{\mathrm{c}} \Big/ \left(\frac{h}{d}\right)_{\mathrm{v}} = a_{\mathrm{bg}} \bar{v} + 1 \qquad \text{(bulged)},$$
$$\left(\frac{h}{d}\right)_{\mathrm{c}} \Big/ \left(\frac{h}{d}\right)_{\mathrm{v}} = a_{\mathrm{bd}} \bar{v} \qquad \text{(budded)}, \qquad (20)$$

with fitting parameter $a_{\mathrm{bd}} = \alpha \left[1 - (1+n)\varphi^{-\frac{1}{2}}\sigma_{\mathrm{c}}\right]^{-1} v_0$. The analytic formula can nicely fit the bulged dataset by setting $a_{\mathrm{bg}} = 0.55$ (Fig. S9D), close to the fitting result of Eq. (19) (i.e. $a_{\mathrm{bg}} = 0.64$), and also fit the budded dataset with $a_{\mathrm{bd}} = 1.4$ (Fig. S9E), the same value with the direct estimation from samples without lumen inflation (Fig. S9C).

To obtain in-plane tension ratio α from the fitting parameters a_{bg} and a_{bd}, we still need to know the normal-state volume v_0 and $(1+n)\varphi^{-\frac{1}{2}}\sigma_{\mathrm{c}}$ (the latter is only for budded organoids). We get the mean value $v_0 = 2.8$ (bulged) and $v_0 = 0.71$ (budded) from the lumen breakage experiment (Fig. 2E), and $(1+n)\varphi^{-\frac{1}{2}}\sigma_{\mathrm{c}} = (1+n)\frac{\epsilon}{2\kappa_{\mathrm{c}}} \approx 0.55$ by using estimation results in Subsection 3.3 and 4.1. This leads to an estimation of $\alpha \approx 1.2$ for bulged organoids, which is consistent with our estimation from samples without lumen inflation (Fig. S9C), as well as the data fitting of thickness ratio from our previous study (Yang et al., 2021). We also obtain $\alpha \approx 0.9$ for budded organoids, close to the estimation for bulged samples.

Overall, this analysis shows that the experimental data can be well-captured both qualitatively and quantitatively (Fig. S9C-E) by our model, and that this can be used to strongly constrain the model parameter – which additionally appear to give consistent values across different datasets and estimation strategies.

3.3. Quantification of differential tension

Here we aim at estimating differential tension – a key parameter in the model. This would allow us to compare experimental data with our theoretical predictions. Before conducting specific measurements, we noticed that the theoretical threshold depends

on crypt size as $\sigma_\mathrm{c} \sim \varphi^{\frac{1}{2}}$ for both the scenarios without and with mechano-sensation (see Subsection 1.2, 2.1 and 2.2 for details). This allows us to combine the effects of differential tension σ_c and crypt size φ, via a new parameter $\varphi^{-\frac{1}{2}}\sigma_\mathrm{c} = \frac{1}{2}\left(\frac{\Gamma_a - \Gamma_b}{\Gamma_l}\right)_\mathrm{c}\sqrt{\frac{4\pi}{N_\mathrm{c}}}$, with N_c the crypt cell number.

To access experimentally this parameter, we separate it into two contributions: (i) the relative difference between apical and basal tension, that is "tension asymmetry" $\epsilon = \frac{\Gamma_a - \Gamma_b}{\Gamma_a + \Gamma_b}$, (ii) the part absent from tension difference, that is $\kappa_\mathrm{c}^{-1} = \left(\frac{\Gamma_a + \Gamma_b}{\Gamma_l}\right)_\mathrm{c}\sqrt{\frac{4\pi}{N_\mathrm{c}}}$. Then the differential tension σ_c used in theory is related to tension asymmetry ϵ as

$$\varphi^{-\frac{1}{2}}\sigma_\mathrm{c} = \frac{\epsilon}{2\kappa_\mathrm{c}}. \tag{21}$$

Tension asymmetry ϵ can be directly estimated from Myosin intensity ratio of apical to basal surfaces. Most intestinal organoids show higher contractility in apical surface than the basal side, thus ϵ is usually in the range of 0 to 1 in our measurements. Quantitatively, we obtain $\epsilon = 0.20/0.21 \pm 0.12$ (mean/median ± SD) for bulged samples, and $\epsilon = 0.44/0.42 \pm 0.08$ for budded ones (Fig. 2F).

The parameter κ_c includes in-plane to lateral tension ratio $\left(\frac{\Gamma_a + \Gamma_b}{\Gamma_l}\right)_\mathrm{c}$, the main regulator of cellular aspect ratio (which we already estimated in Subsection 3.1), and crypt cell number N_c. The cell number in (3D) tissue N_c can be inferred from cell number in (2D) cross-section area n_c (directly counted from sample images, Fig. S9F) by analyzing the geometric parameters of crypt epithelium, including its arclength $l_\mathrm{c} = n_\mathrm{c} d_\mathrm{c}$ and surface area $A_\mathrm{c} = N_\mathrm{c} d_\mathrm{c}^2$. Specifically, bulged crypts have $l_\mathrm{c} = 2R_\mathrm{c}\theta_*$ and $A_\mathrm{c} = \pi(R_\mathrm{c}\theta_*)^2$, which suggests $N_\mathrm{c} = \frac{\pi}{4} n_\mathrm{c}^2$ and $\kappa_\mathrm{c} = \frac{n_\mathrm{c}}{4}\left(\frac{\Gamma_l}{\Gamma_a + \Gamma_b}\right)_\mathrm{c}$; budded crypts have $l_\mathrm{c} = 2\pi R_\mathrm{c}$ and $A_\mathrm{c} = 4\pi R_\mathrm{c}^2$, which leads to $N_\mathrm{c} = \frac{1}{\pi} n_\mathrm{c}^2$ and $\kappa_\mathrm{c} = \frac{n_\mathrm{c}}{2\pi}\left(\frac{\Gamma_l}{\Gamma_a + \Gamma_b}\right)_\mathrm{c}$. Interestingly, the shape factor κ_c is basically unchanged across bulged and budded shapes (Fig. S9G): $\kappa_\mathrm{c} = 0.84/0.75 \pm 0.35$ (bulged, mean/median±SD) and $0.80/0.82 \pm 0.25$ (budded). This allows us to consider κ_c as a constant and focus on the discussion on tension asymmetry ϵ.

4. Quantitative validation of the theory

Here, we test the model by quantitatively comparing its prediction based on theoretically-inferred parameters, such as lumen volume and crypt cellular tensions, with experimental morphogenetic trajectories. We first validate the hypothesis of crypt mechano-sensation, which we had predicted to be important for crypt bistability (Section 2). Based on quantified mechanical parameters (Section 3), we then compare predicted crypt cellular tensions with experimental morphogenetic data: (i) we check if budded samples enter the predicted bistable region; (ii) more quantitatively, we fit theoretical bistable results with measured values of both bulged and budded samples.

4.1. Crypt mechano-sensation

To validate our hypothesis on crypt mechano-sensing, we used lumen inflation experiments as a way to externally change crypt tension/geometry while monitoring the effect on actomyosin levels in crypt and villus. Lumen inflation will simultaneously induce cellular tension changes and deformations in bulged crypts (Fig. 3C). Interestingly, consistent with the curvature or force-sensing mechanism proposed in Section 2, an increase in the radius of curvature or in-plane stretching of the crypt tissue is accompanied by a decrease in apico-basal tension difference. On the other hand, the deformation and cellular tension of budded crypts both keep unchanged with lumen inflation, thus obeying the same tension-deformation relation as bulged crypts (Fig. 3D).

To better validate crypt mechano-sensation, we consider the quantitative relation between cellular tension and tissue geometry should still hold with lumen inflation, i.e. $\epsilon \sim R_{\mathrm{c}}^{-n}$ (or $\lambda_{\mathrm{c}}^{-n}$) in the model (variants of Eqs. (12) and (14)). Although it is convenient to directly link tension difference with geometric/mechanical cues, the calculation of tension difference could bring large errors in data analysis. Instead, we employ tension ratio $z = \Gamma_a/\Gamma_b$ to evaluate the coupling between apico-basal tension difference and tissue curvature/force, and use $\bar{z} = \bar{R}_{\mathrm{c}}^{-b}$ and $\bar{z} = \bar{\lambda}_{\mathrm{c}}^{-b}$ to fit data, with the upper bar denoting parameters normalized by their values before lumen inflation, and b the fitting parameter.

For the curvature-sensation assumption, we get $b = 0.82$ from fitting of mean values of experimental data (Fig. 3E) and $b = 0.78 \pm 0.28$ (best-fit ± 95% confidence interval) from direct fitting of raw data (Fig. S5B). With tension asymmetry $\epsilon = \frac{\Gamma_a - \Gamma_b}{\Gamma_a + \Gamma_b}$ related to tension ratio $z = \frac{\Gamma_a}{\Gamma_b}$ as $\epsilon = \frac{z-1}{z+1}$, the fitted scaling law $z \sim R_c^{-b}$ allows us to infer the relation $\epsilon \sim R_c^{-n}$ used in theory and estimate the sensitivity factor n from parameter b. The pre-factors in these tension-curvature coupling relations can be obtained by using the dataset of budded samples, which shows less fluctuation than that of bulged samples. Then the $\epsilon \sim R_c$ relation can be expressed in two ways: (i) $\epsilon = \frac{z-1}{z+1}$ with $z = z_{bd} \left(\frac{R_c}{R_c^{bd}}\right)^{-b}$; (ii) $\epsilon = \epsilon_{bd} \left(\frac{R_c}{R_c^{bd}}\right)^{-n}$, where z_{bd} and R_c^{bd} are mean values of the budded dataset and $\epsilon_{bd} = \frac{z_{bd}-1}{z_{bd}+1}$ is the corresponding tension asymmetry. The sensitivity factor n can be estimated from b by letting expressions (i) and (ii) in their best fit. Respectively, the fitting of mean values (Fig. 3E) suggests $n = 1.0$, and the direct fitting of raw data (Fig. S5B) estimates $n = 1.0 \pm 0.5$. Thus the two different fitting methods give the same best-fit value ($n = 1.0$).

The same strategy can be applied to determine the scaling law $\epsilon \sim \lambda_c$ in the force-sensation assumption. However, it is more convenient to measure cell height (i.e. epithelium thickness) h_c rather than cell stretch ratio λ_c (characterized by change of cell width, see Subsection 2.2). Using the geometric relation $\lambda_c \sim h^{-\frac{1}{2}}$, we can rewrite the cellular tension – deformation coupling as $\epsilon \sim h_c^{\frac{n}{2}}$ and $z \sim h_c^{\frac{b}{2}}$. Data fitting by $\bar{z} = \bar{h}_c^{\frac{b}{2}}$ (with parameters normalized by their values before lumen inflation) gives $b = 1.57$ (Fig. S6C) and $b = 1.54 \pm 0.54$ (best-fit ± 95% confidence interval, Fig. S6D). Finally, the consistency between $\epsilon = \frac{z-1}{z+1}$ with $z = z_{bd} \left(\frac{h_c}{h_c^{bd}}\right)^{\frac{b}{2}}$ and $\epsilon = \epsilon_{bd} \left(\frac{h_c}{h_c^{bd}}\right)^{\frac{n}{2}}$ suggests $n = 1.6$ (based on fitting the mean values of experimental data in Fig. S6C) and $n = 1.6 \pm 0.7$ (based on direct fitting of raw data in Fig. S6D).

4.2. Threshold for bistability

In the model, the switch from the bulged state (monostable) to bistable state requires the crypt differential tension σ_c to reach a threshold (i.e. the lower bound of crypt bistability analyzed in Subsection 1.2, 2.1 and 2.2). This is because, the apico-basal tension difference in the crypt should be large enough to retain a budded shape even for large lumen volume/stresses (Yang et al., 2021). The prediction that budded samples have tension difference larger than the predicted bistability threshold provides a critical test of the model.

We have obtained analytical thresholds for crypt bistability under various assumptions: Eq. (6) for no sensation, Eq. (13) for curvature-sensation and Eq. (15) for force-sensation. Using the relation between differential tension σ_c and tension asymmetry ϵ (Eq. (21)), and neglecting the volume-related terms in those critical conditions (lumen volume v only weakly impacts the lower bound for bistability, thus here we consider $v \to \infty$ to simplify the expressions), we can rewrite the theoretical threshold for bistability on tension asymmetry ϵ as

$$\begin{aligned}
\epsilon &= \frac{2\kappa_c}{\alpha+1} && \text{(no sensation)},\\
\epsilon &= (1+n)^{-1}\frac{2\kappa_c}{\alpha+1} && \text{(curvature} - \text{sensation)},\\
\epsilon &= \frac{2\kappa_c}{\alpha+1+n} && \text{(force} - \text{sensation)}. \qquad (22)
\end{aligned}$$

Tension asymmetry ϵ of budded samples in Fig. 2F is quantified as $0.44/0.42 \pm 0.08$ (mean/median$\pm$SD). In Eq. (22), we already estimated the in-plane contraction ratio $\alpha \approx 1.2$ (Subsection 3.2) and the parameter $\kappa_c \approx 0.8$ (Subsection 3.3), so the theoretical threshold without mechano-sensation is about 0.73, much higher than the measurement result. However, after taking mechano-sensation into account, we predict the threshold to be 0.36 for curvature-sensation, and 0.42 for force-sensation, both lower values than the experimental data (Fig. 3F, S6E). This quantitative comparison between theoretical threshold and experimental data indicates that the apico-basal tension difference in crypts is large enough for crypt bistability, and mechano-sensation of crypt cells plays a key role in crypt bistability and budding.

4.3. Fitting of bulged and budded samples

Besides the cellular tensions in budded crypts (discussed in Subsection 4.2), the model also predicts cellular tensions in bulged crypts (as bistability theory generally gives two solutions with the same parameter setting). In particular, the model predicts that cellular tensions in bulged and budded crypts have distinct values and change with lumen inflation in different ways (Fig. 3B), in consistent with measurement results (Fig. 3C-D). To further test the model, we directly compared predicted cellular tensions with measured data in both bulged and budded samples. The strategy is to first obtain intrinsic differential tension σ (in Eqs. (12) or (14)) by fitting the budded dataset, then input this value into the model and compare theoretical prediction for bulged crypts with measurement results.

Take the curvature-sensation mechanism as an example, which has intrinsic tension $\sigma = \sigma_c \beta_c^{-n}$ from Eq. (12). For budded crypts, the equilibrium condition $\left.\frac{\partial \hat{F}}{\partial \beta_c}\right|_{\theta_c=0} = 0$ leads to normalized crypt radius $\beta_c \approx \varphi^{\frac{1}{2}} \alpha^{-\frac{1}{3}} \left[1 - (1+n)\varphi^{-\frac{1}{2}}\sigma_c\right]^{\frac{1}{3}}$. Using Eq. (21) to replace differential tension σ_c by tension asymmetry ϵ, we have $\sigma = \frac{\epsilon_{bd}}{2\kappa_c} \varphi^{\frac{n+1}{2}} \alpha^{-\frac{n}{3}} \left[1 - (1+n)\frac{\epsilon_{bd}}{2\kappa_c}\right]^{\frac{n}{3}}$, with ϵ_{bd} the tension asymmetry in budded crypts. In Section 3, we already quantified the tension asymmetry $\epsilon_{bd} = 0.44$ (mean value) and its relevant parameter $\kappa_c = 0.80$, in-plane tension ratio $\alpha = 1.2$ and sensitivity factor $n = 1.0$. The only parameter left is the crypt size φ, which is estimated as $\varphi \approx 0.1$ for both bulged and budded samples (Fig. S9G). Finally, we obtain intrinsic differential tension $\sigma \approx 0.02$, which allows us to nicely fit the budded dataset (Fig. 4C).

Note that the model gives predictions on the dependence of cellular tensions on "normalized lumen volume" v (like those in Fig. 3B), rather than "lumen inflation ratio" $\bar{v}$ quantified in experiments. v and $\bar{v}$ are respectively the ratios of current lumen volumes to that in pressure-free organoids and to initial volume (i.e. volume before lumen inflation). The ratio of initial to free-state volume $v_0 = v/\bar{v}$ can be used for conversion (also used in Subsection 3.2), which can be estimated from the lumen breakage experiment (Fig. 2E): mean value $v_0 = 2.8$ (bulged) and 0.71 (budded).

Importantly, the theoretical predictions can not only fit the measured tension asymmetry ϵ of budded sample, but also agree with the data of bulged samples (Fig. 4C). This provides quantitative evidence that the model can well capture the morpho-mechanics of intestinal organoids in different shapes and developmental stages, and further proves the mechano-sensation of crypt cells.

5. Temporal evolution of crypt morphogenesis

So far, we considered only a quasi-static model, where we assume that crypts are also in mechanical equilibrium, i.e. that model parameters evolve slowly. In this Section, we discuss the possible time-dependence of crypt mechanical parameters. For instance, fate-dependent actomyosin tension could evolve with time independently of lumen volume changes. Besides, mechano-sensation might not be instantaneous, but may need some time to response to curvature or force changes, while Section 2 considers immediate mechano-sensing (see Eq. (12) or (14)).

We extend the model by considering i) the temporal evolution of intrinsic tension and ii) time delays for mechano-sensing. Take curvature-sensation as an example, the crypt differential tension in Eq. (12) now becomes $\sigma_{\rm c} = \sigma(\tau)m(\beta_{\rm c},\tau)$, with $\sigma(\tau)$ the time-related intrinsic tension and $m(\beta_{\rm c},\tau)$ the triggered tension dependent on both the crypt radius β_c and time τ. The temporal evolution of intrinsic tension can be described as

$$\sigma(\tau) = \sigma_{\rm bd} + (\sigma_{\rm bg} - \sigma_{\rm bd})e^{-\tau/\tau_\sigma}, \tag{23}$$

with $\sigma_{\rm bg}$ and $\sigma_{\rm bd}$ respectively the intrinsic tension values of the bulged crypts and budded crypts, τ_σ the characteristic evolution time. The intrinsic tension in budded crypts can be easily estimated as $\sigma_{\rm bd} \approx 0.02$ by direct data fitting in Fig. 4C, which shows high reliability. The temporal evolution of the triggered tension m is assumed to yield

$$\tau_\beta \frac{\partial m}{\partial \tau} = -m + \beta_{\rm c}^{-n}, \tag{24}$$

with τ_β the characteristic time of mechano-sensing.

To mimic the dynamic evolution of crypt morphogenesis, we can also consider a simple evolution law of lumen volume: $v(\tau) = v_0[\bar{v} + (1-\bar{v})e^{-\tau/\tau_v}]$, with τ_v the characteristic time of volume change. Quite similar to Fig. 4A, the theoretical trajectory with temporal effects (Fig. S10A) also highlights the importance of lumen shrinkage during normal crypt morphogenesis, and reproduces the crypt bistable responses to volume inflation. We found such temporal effects would not affect the explanation of experimental observations (for both normal development and perturbation results). Besides, a critical quantitative test of the model is the threshold for crypt budding (Fig. 2F and 3F), which is independent of the intrinsic tension σ (see Eq. (22) in SI Note for details). Thus, whether σ evolves with time or stays constant would not affect the quantitative comparison between the theoretical predictions and experimental measurements in Fig. 2F and 3F.

Regarding possible delays in mechano-sensing, we noticed that the response of myosin levels to the inflation of lumen volume in Fig. 3C and D is quite fast, as the images and data are taken around 15-30min after lumen inflation, which indicates that the timescale of mechano-sensing is also about or even shorter than 30min. This is much faster than cell fate or lumen volume change during morphogenesis, and justifies the quasistatic picture that we had previous adopted. For theoretical completeness, we show how much longer time scales of mechano-sensation would affect organoid dynamics (Fig. S10B-C).

6. Crypt formation in vivo

As in intestinal organoids, crypt morphogenesis in vivo is also driven by cellular tension changes (Sumigray et al., 2018). Gut morphogenesis is also affected by apparent mechanical and geometrical changes in surrounding tissues, such as constriction of the smooth muscle layer in the intestine (Shyer et al., 2013). Furthermore, swelling of villus cells is also observed in vivo (Yang et al., 2021), which could exert forces on crypts in a conceptually similar manner as lumen pressure forces in organoids. Thus, crypt morphogenesis in vivo is likely controlled by two types of forces, i.e. active cellular

forces and external tissue forces generated by surrounding tissues. Here, we explore how our model and findings for closed organoids can be extended to an in vivo geometry.

A crypt in vivo is treated as an open epithelial monolayer with an external force T working at the boundary (see Fig. S10D for schematic). Similar as in the biophysical model for organoids (Subsection 1.1), active cytoskeletal forces in the crypt are captured by the 3D vertex model considering apical, basal and lateral surface tensions. The free energy of the crypt is $F = N_{\mathrm{c}} f_{\mathrm{c}} - T \cdot \pi (R_{\mathrm{c}} \sin\theta_{\mathrm{c}})^2$. To make it easier to compare the similarity and difference between in vivo and in vitro models, we non-dimensionalize the free energy and parameters in the same way with the original model, although simpler dimensionless forms may exist. We obtain the dimensionless free energy $\hat{F} = \frac{F}{\pi(\Gamma_a + \Gamma_b)_{\mathrm{v}} \tilde{R}_0^2}$ as a function of crypt opening angle θ_{c} and dimensionless crypt radius β_{c}:

$$\hat{F}(\theta_{\mathrm{c}}, \beta_{\mathrm{c}}) \approx (\alpha s_{\mathrm{c}} - t \sin^2 \theta_{\mathrm{c}}) \beta_{\mathrm{c}}^2 + \left(16 \varphi^{\frac{3}{2}} s_{\mathrm{c}}^{-\frac{1}{2}} - 8 \varphi \sigma_{\mathrm{c}} \right) \beta_{\mathrm{c}}^{-1}, \qquad (25)$$

where $t = T / (\Gamma_a + \Gamma_b)_{\mathrm{v}}$ is the normalized external force. This free energy (25) is quite similar to the organoid free energy (4), only now the lumen volume-related parameter δ is replaced by the external force t and volume-dependent term $-\frac{8}{3} \delta q_{\mathrm{c}} \beta_{\mathrm{c}}^3 v^{-\frac{1}{3}}$ disappears. This can be explained by the fact that lumen volume/pressure in organoids plays the role as the external tissue force working on the crypt.

In the phase diagram as a function of crypt differential tension σ_{c} and external force t, we can also get a bistable region, which is expanded by mechano-sensing of crypt cells (Fig. S10E). Analytically, for crypt bistability without mechano-sensation, we can easily obtain the lower bound $\sigma_{\mathrm{c}} = \varphi^{\frac{1}{2}} \frac{t}{\alpha + t}$, and the upper bound $\sigma_{\mathrm{c}} = \frac{1}{2} \left(\frac{\varphi}{2} \frac{t}{\alpha - t} \right)^{\frac{1}{2}}$, both are consistent with the numerical results obtained by calculating the local minima of free energy (25) (Fig. S10E). The lower bound becomes $\sigma_{\mathrm{c}} = \frac{\varphi^{\frac{1}{2}}}{1+n} \frac{t}{\alpha + t}$ after considering curvature-sensation (with n the sensitivity factor).

Interestingly, phenomena happened in external tissues, such as constriction of the

smooth muscle layers (Shyer et al., 2013) or enterocyte volume increase on the villus (Yang et al., 2021), would all give rise to compressive forces in the crypt and thus weaken the resistance for crypt budding (i.e. decrease t). Combing these experimental findings with the theoretical phase diagram Fig. S10E, we find that mechanical changes in external tissues (e.g. smooth muscle layer and villus epithelium) are expected to promote crypt budding, while subsequent mechanical perturbations on budded crypts cannot affect the homeostatic state of crypt (stem) cells. Our analysis suggests that the findings in intestinal organoids could also be applied to the in vivo scenario.

References

Shyer, A. E., Tallinen, T., Nerurkar, N. L., Wei, Z., Gil, E. S., Kaplan, D. L., Tabin, C. J., and Mahadevan, L., 2013. Villification: how the gut gets its villi. Science 342: 212–218.

Streichan, S. J., Lefebvre, M. F., Noll, N., Wieschaus, E. F., Shraiman, B. I., 2018. Global morphogenetic flow is accurately predicted by the spatial distribution of myosin motors. Elife, 7: e27454.

Sumigray, K. D., Terwilliger, M., Lechler, T., 2018. Morphogenesis and Compartmentalization of the Intestinal Crypt. Developmental Cell 45: 183–197.

Yang, Q., Xue, S. L., Chan, C. J., Rempfler, M., Vischi, D., Maurer-Gutierrez, F., ... , Liberali, P., 2021. Cell fate coordinates mechano-osmotic forces in intestinal crypt formation. Nature cell biology, 23: 733-744.
