# Supplementary material for: Mechanochemical bistability of intestinal organoids enables robust morphogenesis
Source: Nat Phys. 2025 Feb 28;21(4):608–17. doi: 10.1038/s41567-025-02792-1 (PMC11999871; doi:10.1038/s41567-025-02792-1)
Supplement: Supplementary file 2 — Reporting Summary [file 41567_2025_2792_MOESM2_ESM.pdf]

nature portfolio

Corresponding author(s): Liberali, Hannezo

Last updated by author(s): Jan 8, 2025

Reporting Summary

Nature Portfolio wishes to improve the reproducibility of the work that we publish. This form provides structure for consistency and transparency in reporting. For further information on Nature Portfolio policies, see our Editorial Policies and the Editorial Policy Checklist.

Statistics

For all statistical analyses, confirm that the following items are present in the figure legend, table legend, main text, or Methods section.

n/a	Confirmed	
☐	☒	The exact sample size (*n*) for each experimental group/condition, given as a discrete number and unit of measurement
☐	☒	A statement on whether measurements were taken from distinct samples or whether the same sample was measured repeatedly
☐	☒	The statistical test(s) used AND whether they are one- or two-sided<br>*Only common tests should be described solely by name; describe more complex techniques in the Methods section.*
☒	☐	A description of all covariates tested
☐	☒	A description of any assumptions or corrections, such as tests of normality and adjustment for multiple comparisons
☒	☐	A full description of the statistical parameters including central tendency (e.g. means) or other basic estimates (e.g. regression coefficient) AND variation (e.g. standard deviation) or associated estimates of uncertainty (e.g. confidence intervals)
☒	☐	For null hypothesis testing, the test statistic (e.g. *F*, *t*, *r*) with confidence intervals, effect sizes, degrees of freedom and *P* value noted<br>*Give P values as exact values whenever suitable.*
☒	☐	For Bayesian analysis, information on the choice of priors and Markov chain Monte Carlo settings
☒	☐	For hierarchical and complex designs, identification of the appropriate level for tests and full reporting of outcomes
☒	☐	Estimates of effect sizes (e.g. Cohen's *d*, Pearson's *r*), indicating how they were calculated

Our web collection on statistics for biologists contains articles on many of the points above.

Software and code

Policy information about availability of computer code

Data collection	To collect time-course imaging data, Wako Software Suite (version 2) and custom written segmentation code was used. The code was written in the Liberali lab and implemented with Python 3.8 (relying on multiple open source Python libraries for scientific computing and image analysis). ZEN Black software was used for collecting data of laser nanosurgery. Visview (version 4.4.0.9) was used for collecting confocal imaging data.
Data analysis	To analyze imaging data, Fiji (version 2.9.0) was used. Codes generated and analyzed during the current study are available on https://github.com/fmi-basel/glib-nature_cell_biology2021-materials.git.

For manuscripts utilizing custom algorithms or software that are central to the research but not yet described in published literature, software must be made available to editors and reviewers. We strongly encourage code deposition in a community repository (e.g. GitHub). See the Nature Portfolio guidelines for submitting code & software for further information.

Data

Policy information about availability of data

All manuscripts must include a data availability statement. This statement should provide the following information, where applicable:

- Accession codes, unique identifiers, or web links for publicly available datasets
- A description of any restrictions on data availability
- For clinical datasets or third party data, please ensure that the statement adheres to our policy

All datasets are provided as source Data (excel sheets).

Research involving human participants, their data, or biological material

Policy information about studies with human participants or human data. See also policy information about sex, gender (identity/presentation), and sexual orientation and race, ethnicity and racism.

Reporting on sex and gender	NA
Reporting on race, ethnicity, or other socially relevant groupings	NA
Population characteristics	NA
Recruitment	NA
Ethics oversight	NA

Note that full information on the approval of the study protocol must also be provided in the manuscript.

Field-specific reporting

Please select the one below that is the best fit for your research. If you are not sure, read the appropriate sections before making your selection.

☒ Life sciences ☐ Behavioural & social sciences ☐ Ecological, evolutionary & environmental sciences

For a reference copy of the document with all sections, see nature.com/documents/nr-reporting-summary-flat.pdf

Life sciences study design

All studies must disclose on these points even when the disclosure is negative.

Sample size	No particular statistical method was used to define sample size. In compound time-course experiments, we assumed a minimum of around 50 organoids at Day4 would be sufficient to recognize differences between control and perturbations based upon historical experiments. Sample size was determined based on previous related studies in the field (Yang et al., 2021, Nature Cell Biology).
Data exclusions	Parameters used to exclude datapoints were defined based on data distributions. Exclusion criteria for segmentation were pre-definded as reported previously (Yang et al., 2021, Nature Cell Biology).
Replication	All experiments were replicated at least twice with similar findings.
Randomization	Samples were randomly assigned.
Blinding	Same investigator performed experiments and data analyses therefore it is not blinding.

Reporting for specific materials, systems and methods

We require information from authors about some types of materials, experimental systems and methods used in many studies. Here, indicate whether each material, system or method listed is relevant to your study. If you are not sure if a list item applies to your research, read the appropriate section before selecting a response.

Materials & experimental systems

n/a	Involved in the study
☐	☒ Antibodies
☐	☒ Eukaryotic cell lines
☒	☐ Palaeontology and archaeology
☒	☐ Animals and other organisms
☒	☐ Clinical data
☒	☐ Dual use research of concern
☒	☐ Plants

Methods

n/a	Involved in the study
☒	☐ ChIP-seq
☒	☐ Flow cytometry
☒	☐ MRI-based neuroimaging

Antibodies

Antibodies used

anti-YAP (Cell signaling technology mAB #14074, clone D8H1X, dilution 1:200),
anti-Dll1(R&D Systems AF3970, clone NA (Polyclonal), dilution 1:200),
anti-Lysozyme (DAKO A0099, clone NA (Polyclonal), dilution 1:500),
Alexa Fluor 488 goat anti rabbit IgG (Thermo fisher scientific B40943, dilution 1:2000),
Alexa Fluor 568 donkey anti sheep IgG (Thermo fisher scientific A-21099, dilution 1:2000).

Validation

Validation statements available from manufacturers:
anti- YAP (https://www.cellsignal.com/products/primary-antibodies/yap-d8h1x-xp-rabbit-mab/14074),
anti-Dll1 (https://www.rndsystems.com/cn/products/mouse-rat-dll1-antibody_af3970),
anti-Lysozyme (https://www.labome.com/product/Dako/A0099.html).

Eukaryotic cell lines

Policy information about cell lines and Sex and Gender in Research

Cell line source(s)

Wnt3a L-cells , gift from Novartis.

Authentication

The cell line used was not authenticated.

Mycoplasma contamination

The cell line is tested negative with mycoplasma contamination.

Commonly misidentified lines
(See ICLAC register)

No commonly misidentified lines in this lab.

Plants

Seed stocks

Report on the source of all seed stocks or other plant material used. If applicable, state the seed stock centre and catalogue number. If plant specimens were collected from the field, describe the collection location, date and sampling procedures.

Novel plant genotypes

Describe the methods by which all novel plant genotypes were produced. This includes those generated by transgenic approaches, gene editing, chemical/radiation-based mutagenesis and hybridization. For transgenic lines, describe the transformation method, the number of independent lines analyzed and the generation upon which experiments were performed. For gene-edited lines, describe the editor used, the endogenous sequence targeted for editing, the targeting guide RNA sequence (if applicable) and how the editor was applied.

Authentication

Describe any authentication procedures for each seed stock used or novel genotype generated. Describe any experiments used to assess the effect of a mutation and, where applicable, how potential secondary effects (e.g. second site T-DNA insertions, mosiacism, off-target gene editing) were examined.
